# The SOX10-ACAT2-Cholesterol Synthesis Axis Is Required for Melanoma Proliferation

**DOI:** 10.7150/ijbs.114084

**Published:** 2026-01-15

**Authors:** Lihua Wang, Chenyuan Dai, Jie Yang, Yawen Ma, Xia Ding, Xianqun Fan

**Affiliations:** 1 Department of Cellular and Genetic Medicine, School of Basic Medical Sciences, Fudan University, Shanghai, 200032, China.; 2 Department of Ophthalmology, Shanghai Ninth People's Hospital, Shanghai JiaoTong University School of Medicine, Center for Specialty Strategy Research of Shanghai Jiao Tong University China Hospital Development Institute, Shanghai, China.; 3 Shanghai Key Laboratory of Orbital Diseases and Ocular Oncology, Shanghai, China.

**Keywords:** melanoma, cholesterol biosynthesis pathway, SOX10, ACAT2, TAF15

## Abstract

Research on cholesterol and its metabolic pathways has catalyzed the development of anticancer drugs targeting cholesterol synthesis. However, the cholesterol metabolic state in melanoma remains poorly characterized. In this study, we found that total cholesterol levels and the expression of acetyl-CoA acetyltransferase 2 (ACAT2), a key cholesterogenic enzyme, were significantly elevated in melanoma cells. ACAT2-mediated *de novo* cholesterol synthesis promoted melanoma growth both* in vitro* and *in vivo*. Furthermore, we identified that the transcription factor SOX10, which is critical for melanocyte development, was specifically highly expressed in melanoma and directly upregulated ACAT2 expression, thereby promoting cholesterol synthesis and tumor proliferation. Mechanistically, SOX10 transcriptionally activated ACAT2 expression by interacting with TAF15. This SOX10-TAF15 complex subsequently enhanced ACAT2 protein levels, stimulated cholesterol synthesis, suppressed apoptosis, and ultimately drove melanoma proliferation. Our findings reveal that the SOX10-TAF15-ACAT2 axis is a key regulator of cholesterol synthesis and melanoma proliferation, presenting a promising therapeutic target.

## 1. Introduction

Melanoma originates from the malignant transformation of melanocytes, which are derived from neural crest progenitors 1. Intermittent, intense ultraviolet (UV) exposure is a well-established etiological factor in the development of malignant melanoma 2. The malignant transformation process involves the continuous activation of oncogenic signaling pathways, accompanied by polygenic alterations such as the BRAF-V600E mutation, TERT promoter mutations, CDKN2A inactivation, chromatin-remodeling gene (ARID1A/ARID1B/ARID2) mutations, and mutations in PTEN and TP53^3^, ^4^. Despite these insights, the complete etiology of melanoma remains incompletely understood. Metabolic reprogramming, recognized as one of the hallmarks of cancer 5, not only supports tumor growth by providing energy and biosynthetic precursors but also epigenetically regulates gene expression, thereby profoundly influencing cell fate. Recently, there has been growing interest in lipid metabolism within tumor cells. Lipids are documented to be crucial as structural membrane components, for energy storage, ATP production via oxidation, and as signaling molecules 6-10. Furthermore, our previous study demonstrated that fatty acid synthesis plays a vital role in regulating cellular functions and determining cell fate by modulating mitochondrial fission 11.

Cholesterol synthesis and transport, which are key factors in cancer progression, interact with multiple cancer-related signaling pathways, such as p53, Wnt, and Hedgehog 12-14. Aberrant cholesterol metabolism contributes to tumor development; specifically, enhanced *de novo* cholesterol synthesis in melanoma is directly associated with a poor prognosis 15. These findings underscore the diverse physiological functions of cholesterol and its metabolism, as well as their potential applications in cancer diagnosis and treatment. Pharmacologically targeting this pathway—for instance, by inhibiting HMGCR with statins or targeting other key enzymes and transporters like SQLE, OSC, SOAT1, and LDLR—has demonstrated promising antitumor efficacy 16-18. Reducing cholesterol levels in melanoma cells and T cells within the tumor microenvironment can inhibit metastasis and promote cancer cell death 10, 19. Furthermore, inhibiting cholesterol transport or depleting cholesterol itself impedes melanoma metastasis 20, 21. Despite this evidence, the correlation between cholesterol metabolism and cancer progression remains partially controversial 22, and the specific role of the *de novo* cholesterol synthesis pathway in melanoma progression has not been fully elucidated. Acetyl-CoA acetyltransferase 2 (ACAT2) is a cytoplasmic enzyme that catalyzes the condensation of two acetyl-CoA molecules to form acetoacetyl-CoA. This reaction is the first and rate-limiting step in the mevalonate pathway, which is essential for *de novo* cholesterol synthesis. Subsequent enzymatic reactions, including those catalyzed by HMGCS1 and HMGCR, further process acetoacetyl-CoA to ultimately produce cholesterol. However, the role of ACAT2 in melanoma pathogenesis, specifically whether it regulates melanoma initiation and progression by mediating *de novo* cholesterol synthesis, remains unexplored.

The SOX protein family comprises evolutionarily conserved transcription factors encoded by the Sry-related HMG-box gene. Among them, SOX10 is critical for the establishment and normal function of melanocytes. Through its precise spatio-temporal expression, SOX10 regulates the entire melanocyte life cycle 23, 24. In mammals, the development of the melanoblast lineage is particularly dependent on SOX10 25. While its expression is suppressed in differentiated melanocytes 26, SOX10 is overexpressed in melanoma 27, making it a highly sensitive diagnostic marker for both primary and metastatic lesions 28, 29. However, whether SOX10 regulates cholesterol metabolism in melanoma cells remains unknown.

Our study uncovers the critical role of the *de novo* cholesterol synthesis pathway and its key enzyme, ACAT2, in melanoma progression, and delineates its specific regulatory mechanism. We first observed significant cholesterol accumulation and upregulated expression of *de novo* cholesterogenic enzymes, particularly ACAT2, in melanoma cells, indicating an activated *de novo* synthesis pathway. Functional assays demonstrated that ACAT2-mediated *de novo* cholesterol synthesis is essential for melanoma cell proliferation both *in vitro* and *in vivo*. Furthermore, we identified that the transcription factor SOX10, which is highly expressed in melanoma, directly enhances ACAT2 expression, thereby stimulating cholesterol synthesis and promoting proliferation. Mechanistically, we proved that SOX10 interacts with TAF15 to transcriptionally activate ACAT2 expression. Collectively, our findings reveal a previously unrecognized role of the SOX10-TAF15-ACAT2 axis in reprogramming cholesterol metabolism to drive melanoma progression, presenting a novel therapeutic target.

## 2. Materials and Methods

### Cell culture and reagents

We cultured PIG1, HEK293T, and various melanoma cell lines in Dulbecco's Modified Eagle Medium (DMEM) supplemented with 10% heat-inactivated fetal bovine serum (FBS) and 1% penicillin-streptomycin. DMEM, FBS, and antibiotics were obtained from Thermo Fisher Scientific. Cholesterol (C3045) and filipin III (70440) were sourced from Sigma-Aldrich and Cayman Chemical, respectively. Avasimibe (HY-13215) was acquired from MedChemExpress. The PIG1 human normal melanocyte cell line was kindly provided by the Department of Ophthalmology, Peking University Third Hospital. A375, SK-MEL-28, and HEK293T cells were purchased from the American Type Culture Collection (ATCC). The human conjunctival melanoma cell line CM2005.1 was kindly provided by Prof. Martine J. Jager (Leiden University Medical Center, Leiden, The Netherlands). Human ocular melanoma cell lines OCM1, OCM1a, and OM431 were generously shared by Prof. John F. Marshall (Tumor Biology Laboratory, Cancer Research UK Clinical Centre, London, UK). All cell lines were authenticated by short tandem repeat (STR) profiling.

### Filipin III staining and immunofluorescence

After fixation with 4% paraformaldehyde (PFA) for 30 min at room temperature, cells were washed three times with PBS and stained with 50 μg/mL Filipin III for 45 min. Subsequently, cells were permeabilized with 0.5% Triton X-100 and blocked with 3% bovine serum albumin (BSA). Following blocking, cells were incubated overnight at 4 °C with a primary antibody against histone H2A (2718; Cell Signaling Technology), washed, and then incubated with an appropriate secondary antibody. Nuclei were visualized using the anti-H2A antibody. Images were acquired using a fluorescence microscope.

### Cellular total cholesterol measurement

Cells were lysed in RIPA buffer containing 1% NP-40 (Sangon Biotech) for 45 min. Total cholesterol and protein concentrations in the lysates were quantified using the Biochemical Total Cholesterol Assay Kit (F002-1-1; Nanjing Jiancheng Bioengineering Institute) and the Bradford Assay Kit (C600641; Sangon Biotech), respectively, according to the manufacturers' protocols. Cholesterol levels were normalized to the total protein concentration.

### Quantitative real-time PCR

Total RNA was extracted using TRIzol reagent, and cDNA was synthesized using the iScript cDNA Synthesis Kit (Promega). Quantitative real-time PCR (qPCR) was performed using iQ SYBR Green Supermix on an ABI PRISM 7500 system (Applied Biosystems). Primer sequences are provided in Supplementary Table 3. The relative expression of each transcript was normalized to 18S rRNA, and fold changes in mRNA expression were calculated based on threshold cycle (Ct), where ΔCt = Ct_target_ - Ct_18S_ and Δ(ΔCt) =ΔCt _Control_- ΔCt_ Indicated condition_.

### Western blot analysis

Cells were lysed in RIPA buffer (50 mM Tris-Cl, pH 8.0, 150 mM NaCl, 5 mM EDTA, 0.1% SDS, 1% NP-40). Equal amounts of protein lysates were separated by SDS-PAGE and transferred to PVDF membranes. The following primary antibodies were used: anti-HMGCR, anti-SOAT1 (Abcam), anti-Flag M2 (Sigma-Aldrich), anti-ACAT2, anti-HMGCS1, anti-IDI1, anti-FDPS, anti-ACAT1, anti-SOX10, anti-TAF15, and anti-β-actin (Proteintech). HRP-conjugated anti-rabbit or anti-mouse secondary antibodies (Bio-Rad) were applied, and signals were detected using Western ECL Substrate (Bio-Rad).

### Plasmid construction and RNA interference

The coding sequences (CDS) of ACAT2, SOX10, and TAF15 were cloned into the pSin-EF2-Pur lentiviral vector (Addgene). Short hairpin RNAs (shRNAs) targeting ACAT2, SOX10, and TAF15 were cloned into the pLKO.1 lentiviral vector. Sequences are listed in Supplementary Table 4. Lentiviral particles were produced in HEK293T cells, and SK-MEL-28 and CM2005.1 cells were transduced followed by selection with appropriate antibiotics to generate stable cell lines.

### Apoptosis assay​

Apoptosis was assessed using the FITC Annexin V Apoptosis Detection Kit I (BD Biosciences) according to the manufacturer's protocol. In brief, cells were washed twice with cold PBS, stained with FITC-Annexin V and propidium iodide (PI) on ice for 15 min in the dark, and analyzed on a BD LSRFortessa flow cytometer (BD Biosciences).

### Liquid chromatography-mass spectrometry (LC-MS) metabolite analysis​

Cells were serum-starved for 24 h and then cultured in medium supplemented with 10% FBS and 1 mM sodium acetate-13C₂ (HY-W750791; MedChemExpress) for 48 h. Metabolites were extracted as previously described. Briefly, after collection, cells were flash-frozen in liquid nitrogen and metabolites were extracted using 80% methanol/water with ultrasonication. Protein precipitates were removed by centrifugation. Metabolite separation was performed on an ACQUITY UPLC HSS T3 column (Waters) using a Q Exactive HF mass spectrometer (Thermo Scientific) coupled with a Vanquish UPLC system (Thermo Scientific). The electrospray ionization (ESI) parameters were set as follows. Positive mode: heater temperature 300 °C, sheath gas flow 45 (arb), auxiliary gas flow 15 (arb), sweep gas flow 1 (arb), spray voltage 3.0 kV, capillary temperature 350 °C, S-Lens RF level 30. And negative mode: heater temperature 300 °C, sheath gas flow 45 (arb), auxiliary gas flow 15 (arb), sweep gas flow 1 (arb), spray voltage 3.2 kV, capillary temperature 350 °C, S-Lens RF level 60. Data were processed using Compound Discoverer 3.3 (Thermo Scientific). The enrichment of 13C-labeled metabolites was calculated as the ratio of labeled ion intensity to total ion intensity.

### Chromatin immunoprecipitation (ChIP) assay​

Chromatin immunoprecipitation was performed using the EZ-ChIP Kit (Millipore) according to the manufacturer's instructions. In brief, cells were cross-linked with 1% formaldehyde for 10 min at room temperature, and the reaction was quenched with 125 mM glycine. After collection, SK-MEL-28 cells were sonicated to fragment chromatin. Immunoprecipitation was carried out overnight at 4 °C using either control IgG or an anti-SOX10 antibody (Abcam). Beads were sequentially washed with low-salt, high-salt, LiCl, and TE buffers. DNA was eluted in elution buffer containing proteinase K, reverse cross-linked overnight at 65 °C, and purified using a PCR purification kit. Precipitated DNA was analyzed by quantitative PCR using SYBR Green master mix (Applied Biosystems). ChIP primer sequences are listed in Supplementary Table 5.

### Transfection and luciferase assay

Cells were seeded in 24 well plates. Renilla reporter pRL-TK and pGL2-promoter empty vector, pGL2-ACAT2 promoter or pGL2-ACAT2 mutated promoter were cotransfected with or without pSin-FLAG-SOX10 vector into cells. Twenty-four hours after transfection, the cells were harvested in lysis buffer and subjected to a single freeze-thaw cycle to ensure complete lysis. Cell lysates were transferred to the microcentrifuge tubes, vortexed for 3 min and then centrifuged. Luciferase activity was assessed using the Dual-Luciferase® Reporter Assay System (Promega). Samples were treated as previously described 30. All experiments were repeated at least three times.

### Immunoprecipitation assay

Cells were lysed in lysis buffer (20 mM Tris-HCl pH 8.0, 150 mM NaCl, 2 mM EDTA, 1% NP-40, 1 mM DTT, 1× protease inhibitor cocktails). After sitting on ice for 45 min, the lysates were centrifuged at 4 °C to remove cell debris. Proteins in supernatant were quantified, and equal amount of proteins in supernatant were incubated with anti-FLAG M2, anti-HA, anti-SOX10 or anti-TAF15 antibodies for 4 h, followed by incubation with pre-cleared protein A/G-Sepharose beads for 1 h at 4°C. After incubation, beads were washed three times with lysis buffer, followed by further washing with ice-cold PBS and boiling in 2× loading buffer. Protein samples were resolved by SDS-PAGE. Proteins associated with SOX10 were analysed by mass spectrometry. List of SOX10-interacting candidate proteins from MS analysis are listed in Supplementary Table 2.

### EdU staining

Cells were seeded in 6-well plates and allowed to adhere overnight. Following treatment regimens, they were incubated with 10 μM EdU for 2 hours at 37 °C (duration optimized based on cell doubling time). After labeling, cells were fixed with 4% paraformaldehyde for 15 minutes, washed three times with PBS, and permeabilized using 0.3% Triton X-100 for 15 minutes at room temperature. The Click reaction cocktail was prepared by combining Click Reaction Buffer, CuSO₄, Azide 488, and Click Additive Solution in sequence according to the manufacturer's protocol (Beyotime, C0071S). After removing the washing buffer, cells were incubated with 0.5 mL of the freshly prepared reaction mixture per well for 30 min at room temperature in the dark. Following incubation, cells were washed three times with washing buffer. Nuclei were counterstained with DAPI. The images were captured by immunofluorescence microscopy.

### Animal studies

Animal experiments were approved by the Shanghai Jiao Tong and Fudan University Animal Care and Use Committee and conducted in accordance with institutional guidelines and the National Health and Family Planning Commission of China regulations. For xenograft studies, 5×10⁶ SK-MEL-28 cells stably expressing non-targeting control (NTC) shRNA, shSOX10, ACAT2-overexpressing with or without SOX10 knockdown, or ACAT2-knockdown with or without SOX10 overexpression were subcutaneously injected into 5-week-old male BALB/c nude mice (SJA Laboratory Animal Co., China). Mice were randomly assigned to experimental groups. Tumor volume was measured using digital calipers and calculated as length × width × depth × 0.52. At the endpoint, the mice were humanely killed, and tumors were harvested, photographed, and weighed. Tumor samples were subjected to hematoxylin and eosin (H&E) staining for histology, and immunohistochemical staining for Ki-67 (proliferation) and VEGF (angiogenesis). Flow cytometry was used to analyze the proportion of F4/80⁺CD11b⁺CD206⁺ M2-type tumor-associated macrophages (TAMs). Total cholesterol levels were measured in tumor lysates and normalized by the total protein concentration.

### Immunohistochemical and immunofluorescence Staining

Paraffin-embedded sections from mouse xenografts and human melanoma specimens were baked at 59 °C for 1 h, deparaffinized in xylene, and rehydrated through a graded ethanol series. Antigen retrieval was performed using citrate buffer under microwave heating. Endogenous peroxidase activity was blocked with 3% H₂O₂, and nonspecific binding sites were blocked with 5% BSA. Sections were incubated with primary antibody at 4 °C overnight, followed by HRP-conjugated secondary antibody at 37 °C for 35 min. Signal was developed using a DAB substrate kit, and nuclei were counterstained with hematoxylin. Sections were dehydrated, cleared in xylene, and mounted. For immunofluorescence, sections were stained with antibodies against ACAT2 (MA5-25114; Invitrogen) and SOX10 (10422-1-AP; Proteintech) from normal skin and melanoma tissues (toe, orbit, sole). Nuclei were stained with DAPI. Images were acquired using fluorescence microscopy.

### Statistical analysis

Data are presented as mean ± SD from at least three independent experiments. Statistical significance between two groups was determined using Student's t-test, while comparisons among multiple groups were performed using ANOVA. Kaplan-Meier survival analysis and comparative analysis of ACAT2 expression levels in the TCGA melanoma cohort were conducted through the Protein Atlas platform (https://www.proteinatlas.org/), from which survival curves, box plots, and corresponding log-rank p-values were obtained. Correlation analysis between ACAT2 and SOX10 was performed using expression data from the DFCI database. A p-value ≤ 0.05 was considered statistically significant.

## 3. Results

### Cholesterol synthesis is enhanced in melanoma cells to facilitate cell proliferation

**Figure 1:**
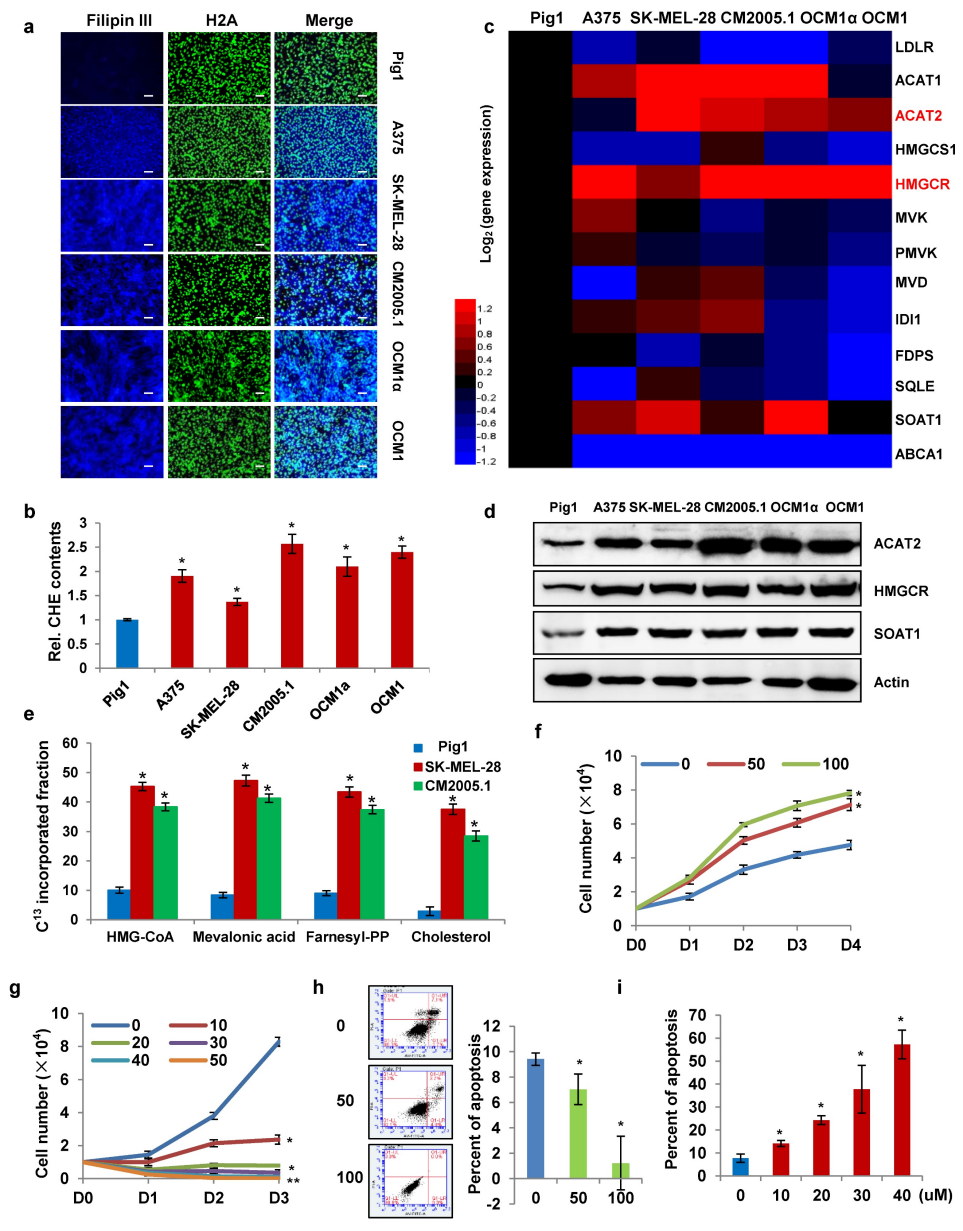
** Enhanced cholesterol synthesis in melanoma cells promotes their proliferation. (a)** Cholesterol in Pig1, A375, SK-MEL-28, CM2005.1, OCM1a, and OCM1 cells was stained with Filipin III. Nuclei were counterstained with H2A. Scale bars: 50 μm. **(b)** Cellular cholesterol content was measured in the indicated cell lines and normalized to total cellular protein. **(c)** Heatmap of qRT-PCR analysis showing mRNA expression of cholesterol metabolism-related genes in A375, SK-MEL-28, CM2005.1, OCM1a, and OCM1 cells relative to Pig1 cells. Red and blue denote upregulation and downregulation, respectively. **(d)** Western blot analysis of ACAT2, HMGCR, and SOAT1 expression in the indicated cell lines. **(e)** LC-MS analysis of ¹³C-labeled HMG-CoA, mevalonic acid, farnesyl-PP, and cholesterol in Pig1, SK-MEL-28, and CM2005.1 cells after 48-hour incubation with 10 mM ¹³C-acetate. **(f)** Growth curves of SK-MEL-28 cells treated with 0, 50, or 100 μg/mL cholesterol, as determined by trypan blue exclusion assay. **(g)** Growth curves of SK-MEL-28 cells treated with 0, 10, 20, 30, 40, or 50 μM avasimibe, as determined by trypan blue exclusion assay. **(h)** Apoptosis analysis of SK-MEL-28 cells treated with 0, 50, or 100 μg/mL cholesterol. **(i)** Apoptosis analysis of SK-MEL-28 cells treated with 0, 10, 20, 30, 40, or 50 μM avasimibe. Data are presented as mean ± SD from three independent experiments. *P < 0.05 compared to the Pig1 group in (b, e) or the 0 group in (f-i) by one-way ANOVA with Bonferroni post-hoc test. Actin was used as a loading control in (d). See also Figure S1.

To investigate the role and underlying mechanisms of cholesterol metabolism and key enzymes in melanoma progression, we compared cholesterol levels in various melanoma cell lines with normal pigment cells. The normal melanocyte Pig1 was used as the control, while the melanoma cell lines included A375, SK-MEL-28, CM2005.1, OCM1α, and OCM1. First, intracellular cholesterol was stained with Filipin III and observed under a fluorescence microscope. Compared to the control, cholesterol content was significantly elevated in all melanoma cell lines (Fig. 1a). Consistently, measurements using a total cholesterol assay kit confirmed that melanoma cells contained markedly higher cholesterol levels than normal melanocytes (Fig. 1b). These findings indicate that cholesterol content is higher in melanoma cells than in normal melanocytes. We also investigated the metabolic pathways leading to cholesterol accumulation in melanoma cells. Reverse transcription quantitative real-time PCR (qRT-PCR) was performed to evaluate the mRNA expression of enzymes involved in cholesterol uptake (LDLR), efflux (ABCA1), and *de novo* synthesis (ACAT2, HMGCS1, HMGCR, MVK, PMVK, MVD, IDI1, FDPS, SQLE, SOAT1) (Fig. 1c). The full names and functional annotations of these enzymes are detailed in Supplementary Table 1, while their specific metabolic roles have been comprehensively elaborated in our previous paper 31. The results showed that LDLR and ABCA1 expression was significantly suppressed in melanoma cells, suggesting reduced cholesterol exchange with the microenvironment. In contrast, the expression of ACAT2, HMGCR, and SOAT1 was markedly upregulated compared to normal melanocytes. These findings were validated by Western blot (Fig. 1d).

Furthermore, we performed metabolic labeling using ^13^C-acetate to track the incorporation of the isotope into newly synthesized cholesterol. In addition to quantifying *de novo* cholesterol levels, we also detected newly synthesized intermediates in the cholesterol biosynthesis pathway, including HMG-CoA, mevalonic acid, and farnesyl pyrophosphate. LC-MS analysis revealed that the isotopic enrichment of metabolites in the *de novo* synthesis pathway was significantly higher in SK-MEL-28 and CM2005.1 melanoma cells compared to Pig1 melanocytes (Fig. 1e). The elevated incorporation of ^13^C into cholesterol and its intermediates indicates an enhanced rate of cholesterol synthesis in melanoma cells. Overall, these results demonstrate that melanoma cells exhibit an enhanced *de novo* cholesterol synthesis pathway compared to normal melanocytes.

To determine whether cholesterol accumulation is a consequence or a driver of tumorigenesis, we simulated cholesterol accumulation and depletion states in an* in vitro* culture system. Exogenous cholesterol was added to the culture medium to mimic intracellular cholesterol elevation. We found that cholesterol supplementation promoted the proliferation of SK-MEL-28 cells (Fig. 1f). Conversely, treatment with avasimibe, a small-molecule inhibitor of SOAT1, reduced total cholesterol levels in SK-MEL-28 cells (Supplementary Fig. S1a) and suppressed cell proliferation in a dose-dependent manner (Fig. 1g). Previous studies have shown that elevated intracellular cholesterol inhibits the release of mitochondrial pro-apoptotic factors, thereby promoting cancer cell survival 15, 32. In addition, cholesterol enrichment enhances PI3K/AKT signaling by increasing lipid raft formation at the plasma membrane. Lipid rafts are critical for TNF-induced NF-κB activation, which further contributes to apoptosis inhibition 33, 34. Consistent with these reports, exogenous cholesterol supplementation similarly suppressed apoptosis in SK-MEL-28 cells (Fig. 1h). In contrast, avasimibe-induced cholesterol depletion increased the apoptosis rate in a dose-dependent manner (Fig. 1i). These results confirm that cholesterol accumulation promotes melanoma cell proliferation by inhibiting apoptosis.

### SOX10 enhances cholesterol synthesis via ACAT2 in melanoma cells

**Figure 2:**
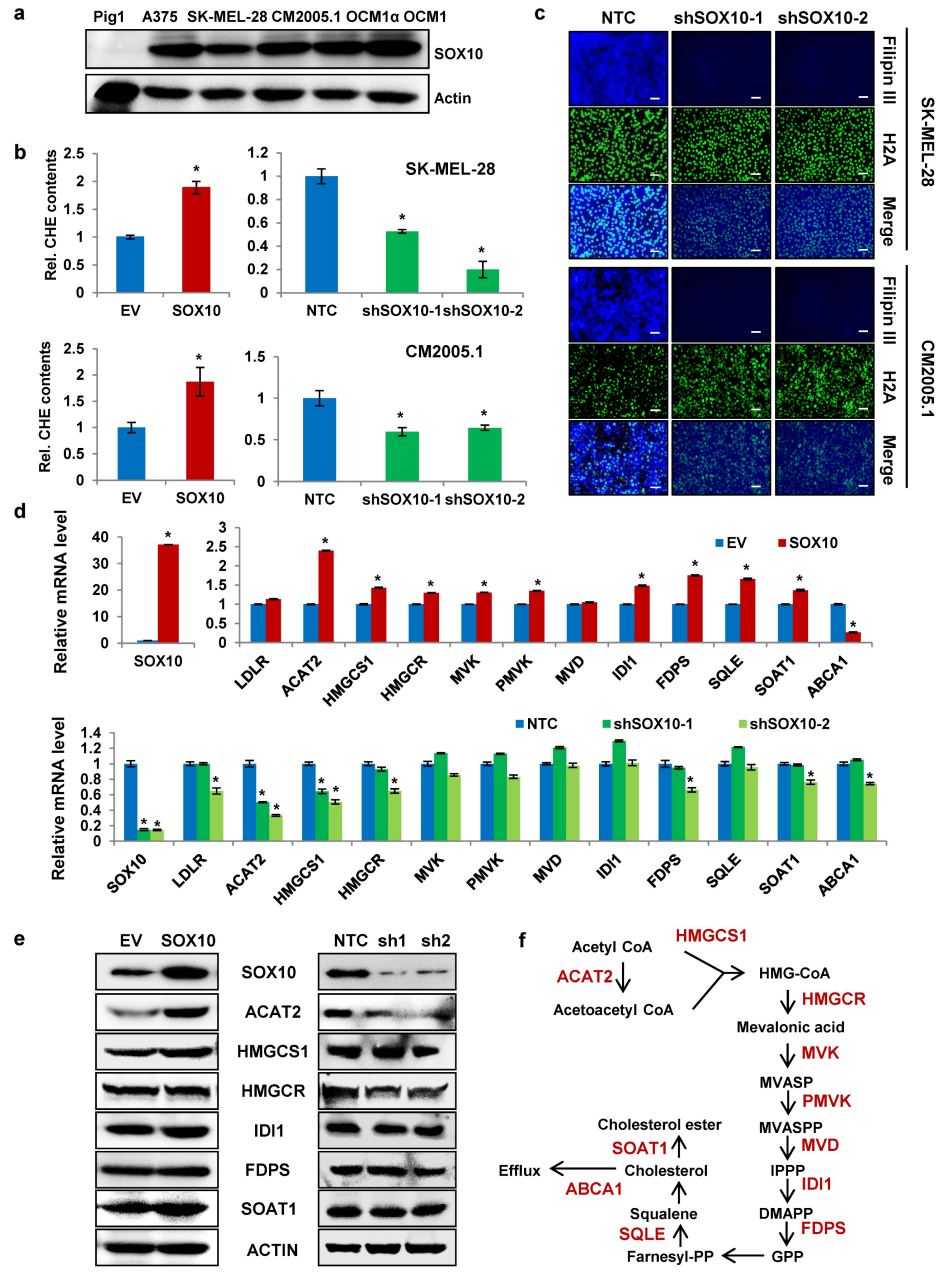
** SOX10 enhances cholesterol synthesis via ACAT2 in melanoma cells. (a)** Western blot analysis of SOX10 expression in Pig1, A375, SK-MEL-28, CM2005.1, OCM1a, and OCM1 cells. **(b)** Cellular cholesterol content was measured in SK-MEL-28 and CM2005.1 cells stably overexpressing or with knocked-down SOX10, normalized to total cellular protein. **(c)** Cholesterol staining with Filipin III in SK-MEL-28 and CM2005.1 cells following SOX10 knockdown. Nuclei were counterstained with H2A. Scale bars: 50 μm. **(d)** qRT-PCR analysis of cholesterol metabolism-related genes in SK-MEL-28 cells stably overexpressing or with knocked-down SOX10. **(e)** Western blot analysis of ACAT2, HMGCS1, HMGCR, IDI1, FDPS, and SOAT1 expression in SK-MEL-28 cells with SOX10 overexpression or knockdown. **(f)** Schematic diagram of the cholesterol synthesis pathway. Data are shown as mean ± SD from three independent experiments. *P < 0.05 versus the EV or NTC group in (b, d) by one-way ANOVA with Bonferroni post-hoc test. Actin was used as a loading control in (a, e). See also Figure S2.

SOX10 is a transcription factor that plays a critical role in melanocyte development 24, 26 and is highly expressed in melanoma 27. In this study, SOX10 protein expression was significantly elevated in multiple melanoma cell lines compared with the normal melanocyte line PIG1 (Fig. 2a). To determine whether SOX10 influences cholesterol levels in melanoma cells, we established stable SOX10-overexpressing and SOX10-knockdown SK-MEL-28 and CM2005.1 cell lines. Western blot confirmed successful generation of these cell lines (Supplementary Fig. S2a). Total cholesterol measurements showed that SOX10 overexpression increased cellular cholesterol content in both melanoma lines, whereas SOX10 knockdown significantly reduced intracellular cholesterol levels (Fig. 2b). Filipin III staining further revealed decreased cholesterol intensity upon SOX10 knockdown (Fig. 2c), indicating that SOX10 positively regulates cholesterol content in melanoma cells.

To explore how SOX10 modulates cholesterol metabolism, we evaluated mRNA expression of cholesterol-related genes (Fig. 2f and Supplementary Table 1) in SK-MEL-28 cells with altered SOX10 expression. SOX10 positively regulated the mRNA levels of ACAT2, HMGCS1, HMGCR, IDI1, FDPS, and SOAT1. Among these, ACAT2 exhibited the most pronounced change in response to SOX10 modulation (Fig. 2d). Consistent with this, SOX10 also enhanced ACAT2 protein expression (Fig. 2e). Similar regulatory trends were observed in CM2005.1 cells. SOX10 overexpression upregulated mRNA levels of ACAT2, HMGCS1, HMGCR, IDI1, FDPS, and SOAT1 (Supplementary Fig. S2b), while SOX10 knockdown suppressed their expression (Supplementary Fig. S2c). Protein analysis in CM2005.1 cells confirmed that ACAT2 levels correlated with SOX10 expression (Supplementary Fig. S2d). Together, these results demonstrate that SOX10 promotes cholesterol accumulation in melanoma cells primarily through transcriptional regulation of ACAT2.

### SOX10-TAF15 interactions are essential for ACAT2 transcriptional expression

**Figure 3:**
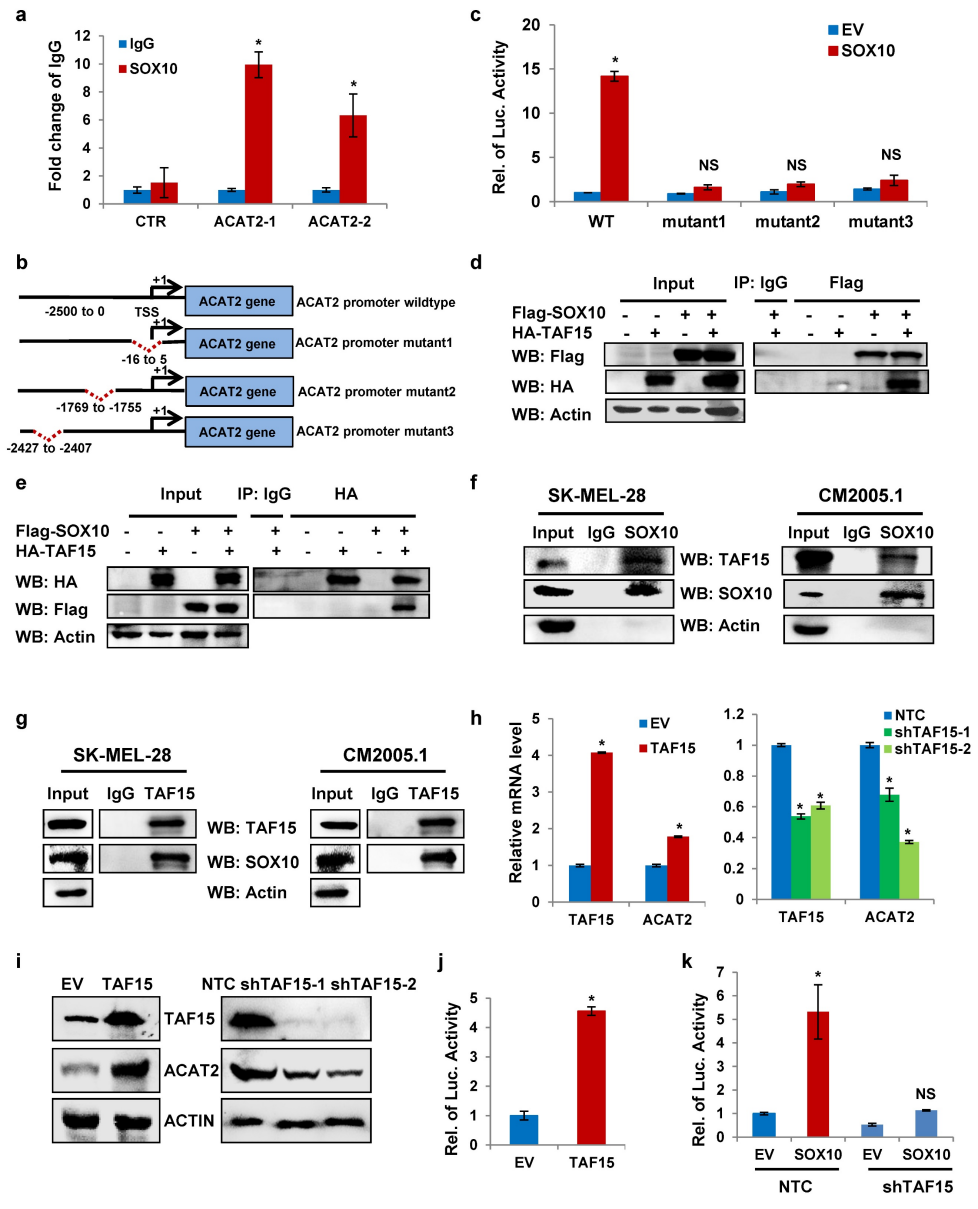
** The SOX10-TAF15 interaction drives ACAT2 transcriptional activation. (a)** ChIP-qPCR analysis of SOX10 binding to potential sites in the ACAT2 promoter in SK-MEL-28 cells using anti-SOX10 or IgG antibody. **(b)** Schematic diagram of promoter mutation sites. **(c)** Luciferase activity of wild-type or mutant ACAT2 promoter reporters in SK-MEL-28 cells expressing empty vector (EV) or SOX10. **(d-g)** Co-immunoprecipitation assays in HEK293T cells co-transfected with Flag-SOX10 and HA-TAF15** (d, e)** or in SK-MEL-28 and CM2005.1 cells** (f, g),** using equal protein amounts for immunoprecipitation with the indicated antibodies, followed by immunoblotting with anti-HA** (d)**, anti-Flag **(e)**, anti-TAF15** (f)**, or anti-SOX10 **(g)** antibody. **(h)** QRT-PCR analysis of ACAT2 expression in SK-MEL-28 cells with TAF15 overexpression or knockdown. **(i)** Western blot analysis of ACAT2 and TAF15 expression in SK-MEL-28 cells with TAF15 overexpression or knockdown. **(j)** Luciferase activity of the ACAT2 promoter reporter in SK-MEL-28 cells expressing EV or TAF15. **(k)** Luciferase activity of the ACAT2 promoter reporter in SOX10-overexpressing SK-MEL-28 cells further infected with shTAF15 or non-targeting control (NTC) virus. Data are presented as mean ± SD from three independent experiments. *P < 0.05 compared to the indicated control groups in each panel by one-way ANOVA with Bonferroni post-hoc test. Actin served as a loading control in (d-g, i). See also Figure S3.

Our previous experiments demonstrated that SOX10 upregulates ACAT2 expression at the mRNA level. To determine whether SOX10 regulates ACAT2 transcriptionally, we performed chromatin immunoprecipitation quantitative PCR (ChIP-qPCR) in SK-MEL-28 cells. The results showed that SOX10 binds to the ACAT2 promoter region (Fig. 3a). The consensus DNA-binding motif recognized by SOX proteins is C(T/A)TTTG(T/A)(T/A) 35, 36. Based on the SOX-recognition motif, we predicted three binding sites in the ACAT2 promoter and introduced site-directed mutations 37-39: Mutation 1 (-16 to +5 bp, TTTCAATATGTCAAATTTGAT→TCGGCGGATGGGCGGCCGCGG), Mutation 2 (-1769 to -1755 bp, CAAAACAATGTAAA→CAACGGCGGTTAAA), and Mutation 3 (-2427 to -2407 bp, CTGTTTGGCTTTTGTTAT→CGGCGGTTGGCTCCGCGTAT) (Fig. 3b). Dual-luciferase reporter assays in HEK293T cells showed that SOX10 overexpression enhanced luciferase activity driven by the wild-type ACAT2 promoter, but had no significant effect when any of the three mutated promoter constructs were used (Fig. 3c). These results indicate that SOX10 binds to the predicted sites in the ACAT2 promoter and enhances its transcriptional activity.

To identify proteins involved in SOX10-mediated regulation of ACAT2, we performed co-immunoprecipitation followed by mass spectrometry Supplementary Table 2, which revealed an interaction between SOX10 and TAF15 (TATA-binding protein-associated factor 15), a transcriptional activator that binds promoter regions of target genes 40-42. We hypothesized that SOX10 cooperates with TAF15 to enhance ACAT2 transcription in melanoma cells. To validate this interaction, we expressed FLAG-tagged SOX10 and HA-tagged TAF15 in HEK293T cells. Co-immunoprecipitation assays confirmed their binding, as anti-FLAG antibody pulled down HA-TAF15 (Fig. 3d) and anti-HA antibody pulled down FLAG-SOX10 (Fig. 3e). We next examined endogenous interactions in SK-MEL-28 and CM2005.1 cells. Immunoblotting detected TAF15 in SOX10 pull-downs (Fig. 3f) and SOX10 in TAF15 pull-downs (Fig. 3g), demonstrating endogenous binding between SOX10 and TAF15. Stable SK-MEL-28 and CM2005.1 cell lines with overexpressed or knocked-down TAF15 were used to validate the regulation of ACAT. TAF15 overexpression increased ACAT2 mRNA levels, while its knockdown reduced them (Fig. 3h). Similarly, ACAT2 protein levels were positively regulated by TAF15 in SK-MEL-28 (Fig. 3i) and CM2005.1 (Supplementary Fig. S3a) stable cells. Dual-luciferase reporter assay in HEK293T cells further demonstrated that TAF15 enhances ACAT2 promoter activity (Fig. 3j).

To determine whether SOX10 requires TAF15 to regulate ACAT2 expression, we examined the effect of SOX10 overexpression on ACAT2 in TAF15-knockout and control SK-MEL-28 cells. QRT-PCR (Supplementary Fig. S3b-d) and Western blot (Supplementary Fig. S3e) data showed that the positive regulation of ACAT2 by SOX10 was significantly attenuated in TAF15-knockout cells compared to controls, indicating that TAF15 is essential for SOX10-mediated ACAT2 upregulation. Moreover, the luciferase experiment data also showed that TAF15 knockdown abolished the SOX10-induced increase in ACAT2 expression (Fig. 3k). Together, these results suggest that SOX10 depends on TAF15 to regulate ACAT2 expression.

### SOX10/TAF15-mediated ACAT2 expression and cholesterol synthesis are critical for melanoma cell proliferation

**Figure 4:**
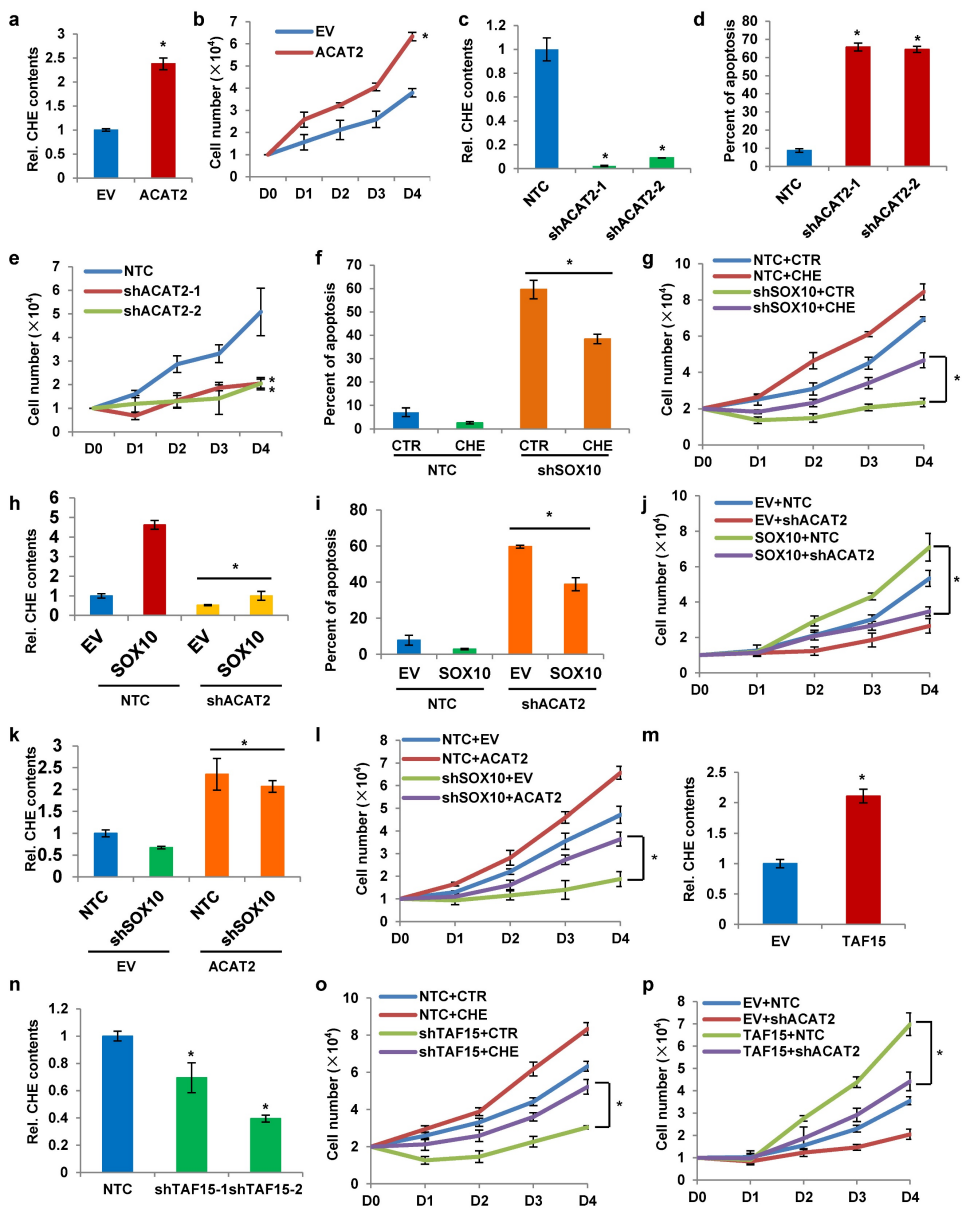
** The SOX10/TAF15 complex promotes cholesterol synthesis and cell proliferation via ACAT2 in melanoma cells. (a)** Cellular cholesterol content in SK-MEL-28 cells overexpressing ACAT2, normalized to total protein. **(b)** Growth curves of ACAT2-overexpressed SK-MEL-28 cells. **(c)** Cellular cholesterol content in SK-MEL-28 cells expressing shACAT2, normalized to total protein. **(d)** Apoptosis analysis of ACAT2-knockdown SK-MEL-28 cells. **(e)** Growth curves of ACAT2-knockdown SK-MEL-28 cells. **(f, g)** Apoptosis** (f)** and cell growth** (g)** in SOX10-knockdown SK-MEL-28 cells supplemented with cholesterol. **(h)** Cellular cholesterol content in SOX10-overexpressing SK-MEL-28 cells with ACAT2 knockdown, normalized to total protein. **(i, j)** Apoptosis** (i)** and cell growth** (j)** in SOX10-overexpressing SK-MEL-28 cells with ACAT2 knockdown. **(k)** Cellular cholesterol content in SOX10-knockdown SK-MEL-28 cells overexpressing ACAT2, normalized to total protein. **(l)** Growth curves of SOX10-knockdown SK-MEL-28 cells overexpressing ACAT2. **(m, n)** Cellular cholesterol content in SK-MEL-28 cells with TAF15 overexpression** (m)** or knockdown **(n)**, normalized to total protein. **(o, p)** Growth curves of TAF15-knockdown SK-MEL-28 cells supplemented with cholesterol** (o)**, and of TAF15-overexpressing SK-MEL-28 cells with ACAT2 knockdown **(p).** Data are shown as mean ± SD from three independent experiments. *P < 0.05 versus the indicated control groups by one-way ANOVA with Bonferroni post-hoc test.

To investigate the functional role of ACAT2 in melanoma, we established an ACAT2-overexpressing SK-MEL-28 stable cell line. Total cholesterol content was significantly increased in these cells (Fig. 4a), accompanied by an enhanced proliferation rate (Fig. 4b). Conversely, ACAT2 knockdown using two independent shRNAs reduced intracellular cholesterol levels (Fig. 4c), increased apoptosis (Fig. 4d), and suppressed cell proliferation (Fig. 4e). These results indicate that ACAT2 promotes cholesterol accumulation, inhibits apoptosis, and enhances proliferation in melanoma cells.

We next examined whether cholesterol supplementation could counteract the effects of SOX10 knockdown. Exogenous cholesterol addition to SOX10-knockdown SK-MEL-28 cells attenuated apoptosis (Fig. 4f) and restored cell proliferation (Fig. 4g). In SK-MEL-28 melanoma cells with SOX10 knockdown, rescue expression of ACAT2 markedly increased the levels of nascent metabolites in the cholesterol synthesis pathway that were suppressed by SOX10 depletion (Supplementary Fig. S4a). Conversely, when ACAT2 was knocked down under SOX10 overexpression conditions, the increase in nascent metabolite levels induced by SOX10 was abolished (Supplementary Fig. S4b). These results indicate that ACAT2 acts downstream of SOX10 to regulate the *de novo* cholesterol synthesis pathway in melanoma cells. Furthermore, ACAT2 knockdown in SOX10-overexpressing cells suppressed the SOX10-induced increase in cholesterol content (Fig. 4h), reduction in apoptosis (Fig. 4i), and acceleration of proliferation (Fig. 4j). Conversely, ACAT2 overexpression in SOX10-knockdown cells rescued the decreased cholesterol levels (Fig. 4k) and impaired proliferation (Fig. 4l) resulting from SOX10 deficiency. Together, these data demonstrate that ACAT2 acts downstream of SOX10 to regulate cholesterol metabolism and melanoma cell growth.

Our previous findings indicated that SOX10 interacts with TAF15 to form a transcriptional complex that activates ACAT2 expression. We therefore hypothesized that TAF15 would exert effects on cholesterol metabolism and cell proliferation similar to those of SOX10. Consistent with this, TAF15 overexpression increased intracellular cholesterol content (Fig. 4m), while TAF15 knockdown reduced it (Fig. 4n). Supplementing cholesterol in the culture medium of TAF15-knockdown SK-MEL-28 cells restored the proliferation impaired by TAF15 depletion (Fig. 4o). Moreover, ACAT2 knockdown attenuated the enhanced proliferation induced by TAF15 overexpression (Fig. 4p). Protein expression levels for the cell lines used in Fig. 4h-p are provided in Supplementary Fig. S4c-e. To further validate the functional role of the SOX10/TAF15/ACAT2 axis, we performed EdU incorporation assays. SOX10 overexpression increased proliferation, which was suppressed by ACAT2 knockdown (Supplementary Fig. S4f). Conversely, under SOX10-knockdown conditions, ACAT2 overexpression rescued cell proliferation (Supplementary Fig. S4g). Similarly, ACAT2 knockdown abrogated the pro-proliferative effect of TAF15 overexpression (Supplementary Fig. S4h). Together, these results demonstrate that the SOX10-TAF15 complex was found to affect intracellular cholesterol content by controlling ACAT2 expression, which further regulates apoptosis and proliferation of melanoma cells.

### ACAT2 is essential for SOX10-mediated melanoma proliferation *in vivo*

**Figure 5:**
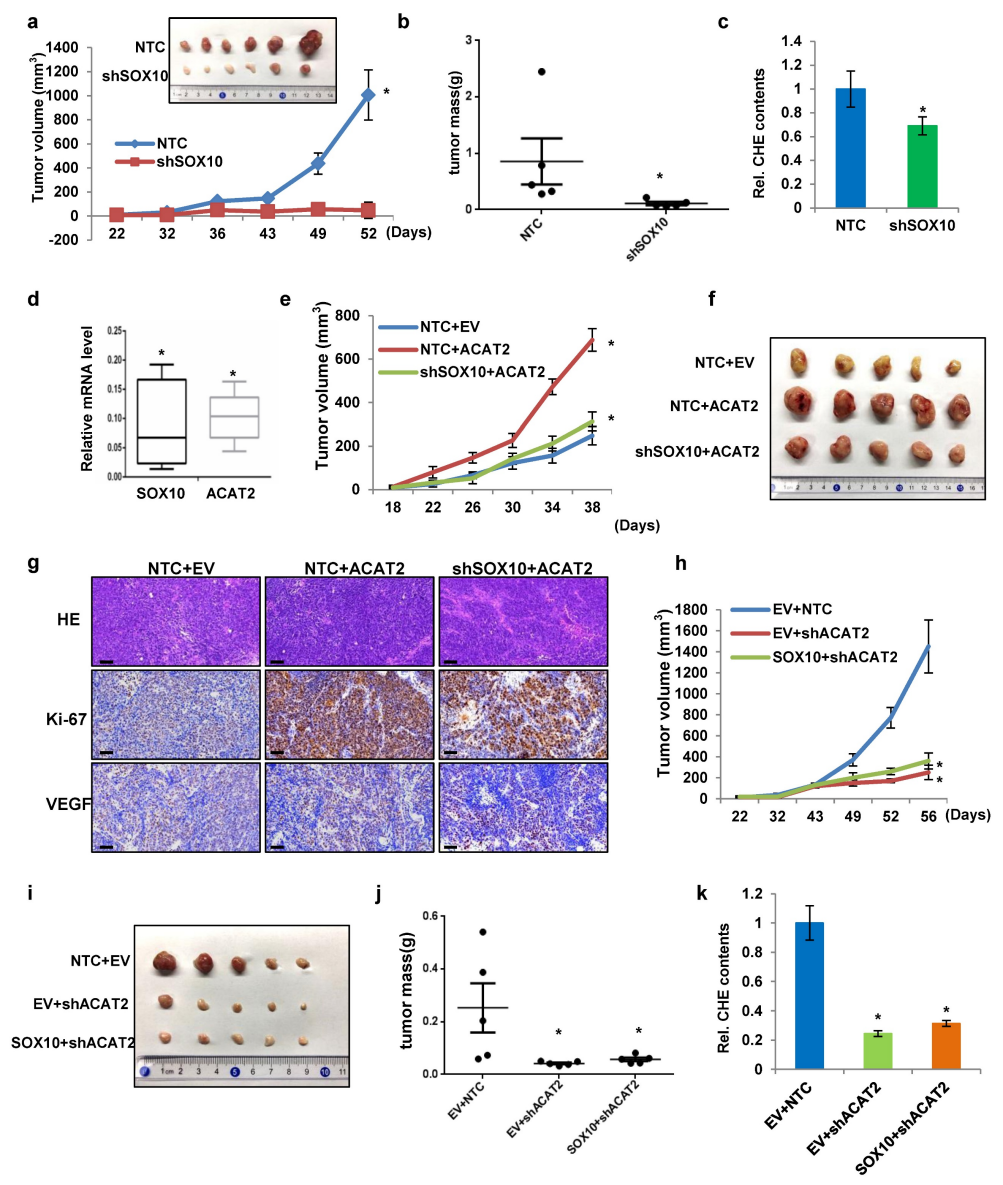
** ACAT2 is essential for SOX10-mediated melanoma proliferation *in vivo*. (a)** SK-MEL-28 cells stably expressing NTC or shSOX10 were subcutaneously injected into nude mice (n = 6 per group). Tumor growth was monitored from day 22 post-inoculation. Tumors were excised and photographed at endpoint. **(b)** Tumor weights from five independent tumors per group in (a). **(c)** Cellular cholesterol content in six independent tumors per group from (a), normalized to total protein. **(d)** SOX10 and ACAT2 mRNA levels measured by qRT-PCR in tumors from (a), normalized to the NTC group. **(e, f)** SK-MEL-28 cells expressing NTC or shSOX10 with ACAT2 overexpression were injected into nude mice (n = 5 per group). Tumor growth curves** (e)** and representative images **(f)** are shown. **(g)** HE staining, Ki-67 (proliferation), and VEGF (angiogenesis) immunohistochemistry of tumor sections from** (e, f)**. **(h-j)** SK-MEL-28 cells with ACAT2 knockdown, with or without SOX10 overexpression, were injected into nude mice (n = 5 per group). Tumor growth curves** (h)**, representative images** (i)**, and weights** (j)** are shown. **(k)** Cellular cholesterol content in five independent tumors per group from (h-j), normalized to total protein. Data represent mean ± SD. *P < 0.05 versus the NTC group in (a-d) or versus the indicated control groups in (e-k) by one-way ANOVA with Bonferroni post-hoc test.

Having shown that SOX10 promotes melanoma proliferation *in vitro* via ACAT2-driven cholesterol synthesis, we next examined whether this regulatory axis operates* in vivo*. We subcutaneously injected BALB/c nude mice with SK-MEL-28 cells stably expressing either non-targeting control (NTC) or SOX10-targeting shRNA. SOX10 knockdown significantly reduced tumor growth (Fig. 5a). At the endpoint, the subcutaneous tumors were collected and analyzed. SOX10-deficient tumors showed smaller size (Fig. 5a) and lower weight (Fig. 5b) compared to NTC. Histological and immunohistochemical analyses of tumor sections revealed that SOX10 knockdown increased apoptosis and decreased proliferation (Supplementary Fig. S5a). Flow cytometry of freshly isolated tumors showed a significant reduction in F4/80⁺CD11b⁺CD206⁺ M2-type tumor-associated macrophages (TAMs) upon SOX10 knockdown (Supplementary Fig. S5b). Total cholesterol levels were also lower in shSOX10 tumors (Fig. 5c). Moreover, qRT-PCR analysis indicated that SOX10 and ACAT2 mRNA levels in shSOX10 tumors were markedly downregulated (0.07 and 0.11, respectively) relative to the NTC group (normalized to 1) (Fig. 5d). These results demonstrate that SOX10 regulates ACAT2 expression and cholesterol accumulation within the tumor microenvironment to promote melanoma growth *in vivo.*

Next, to determine whether ACAT2 and the SOX10-ACAT2 regulatory axis function similarly *in vivo*, we overexpressed ACAT2 in SK-MEL-28 cells expressing either non-targeting control (NTC) or SOX10-targeting shRNA. ACAT2 overexpression restored the *in vivo* proliferation of tumor cells suppressed by SOX10 knockdown (Fig. 5e). Excised tumors showed corresponding changes in morphology (Fig. 5f) and weight (Supplementary Fig. S5c), confirming ACAT2's role in promoting tumor growth. ACAT2 overexpression also increased intratumoral cholesterol content and rescued the cholesterol reduction caused by SOX10 knockdown (Supplementary Fig. S5d). Immunohistochemical analysis indicated that ACAT2 overexpression reversed SOX10 knockdown-induced apoptosis and restored proliferation (Fig. 5g). VEGF staining suggested that SOX10 knockdown reduced VEGF expression, which was partially—though not significantly—restored by ACAT2. Flow cytometry revealed that ACAT2 overexpression alone increased M2-type tumor-associated macrophage (TAM) infiltration, and rescued the reduction in M2 TAMs caused by SOX10 knockdown (Supplementary Fig. S5e).

Conversely, the *in vivo* tumor growth curves showed that ACAT2 knockdown significantly inhibited melanoma cell proliferation, regardless of SOX10 overexpression (Fig. 5h). At the end of the xenograft experiments, Tumors from ACAT2-knockdown groups showed reduced size (Fig. 5i) and weight (Fig. 5j). Moreover, ACAT2 knockdown significantly inhibited tumor size and weight independently of SOX10 overexpression. ACAT2 knockdown also decreased intratumoral cholesterol levels independently of SOX10 status (Fig. 5k). Histopathological assessment confirmed that ACAT2 knockdown enhanced apoptosis and suppressed proliferation, effects not reversed by SOX10 overexpression (Supplementary Fig. S5f). It is worth noting that ACAT2 knockdown substantially decreased VEGF expression, and this decrease was not rescued by SOX10 overexpression. These results further support the conclusion that ACAT2 acts downstream of SOX10 in modulating VEGF protein levels. And ACAT2 knockdown decreased M2-type TAM infiltration *in vivo*, and this effect was not rescued by SOX10 overexpression Supplementary Fig. 5Sg). These findings indicate that ACAT2 acts downstream of SOX10 to promote melanoma growth *in vivo* by enhancing the recruitment of TAMs.

In conclusion, these results demonstrate that ACAT2 affects cholesterol synthesis downstream of SOX10 in melanoma cells and also affects the *in vivo* tumor proliferation.

### SOX10-ACAT2 Axis Is Associated with Poor Prognosis in Human Melanoma​

**Figure 6:**
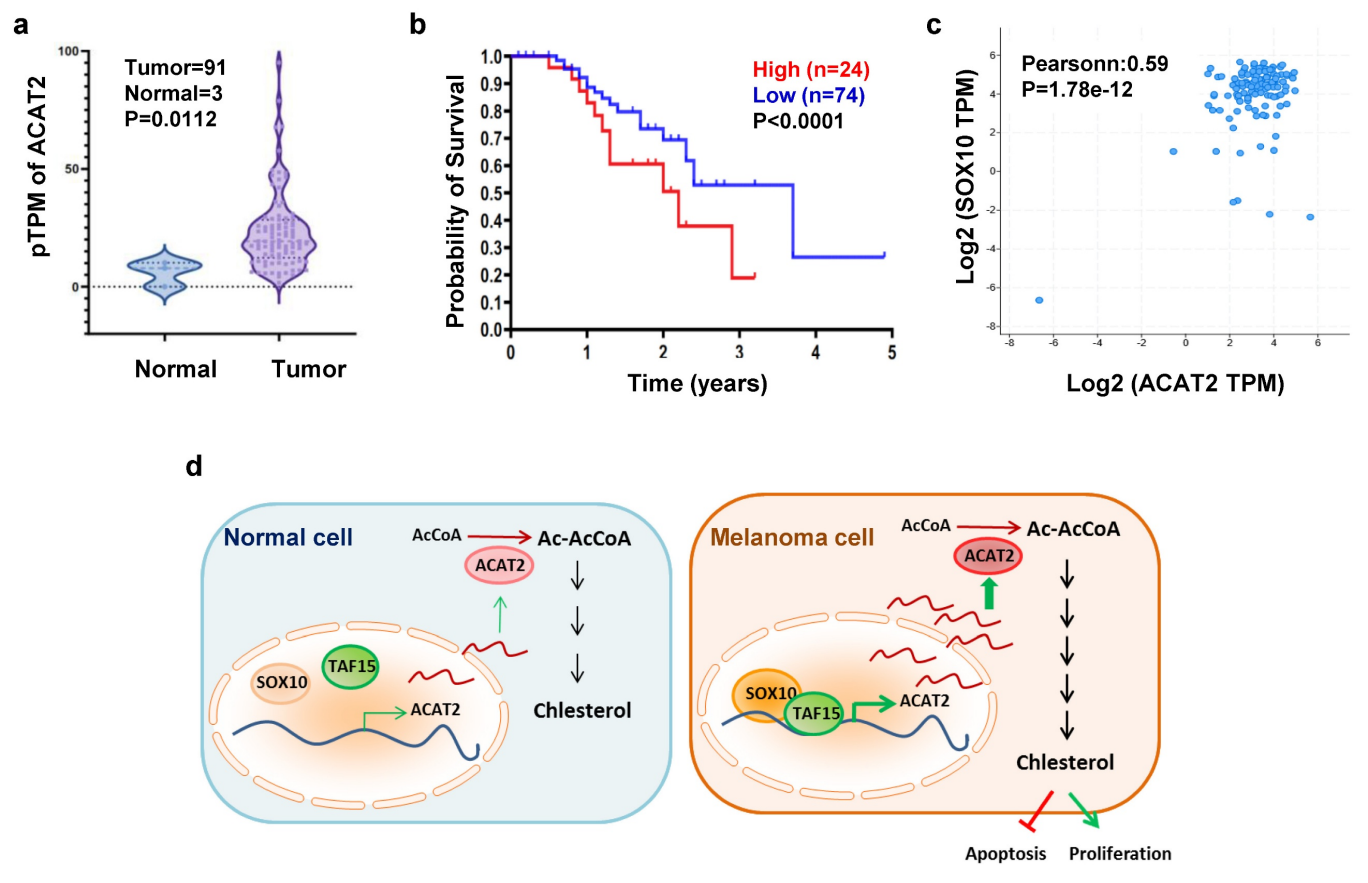
** The SOX10-ACAT2 axis correlates with poor prognosis in human melanoma. (a)** Transcripts per million (TPM) of ACAT2 in melanoma and normal tissue samples from the TCGA database. **(b)** Kaplan-Meier survival analysis of patients stratified by low vs. high ACAT2 expression. **(c)** Analysis of melanoma samples from the DFCI database revealed a correlation between ACAT2 and SOX10 expression. **(d)** Proposed model: The SOX10-TAF15 complex transcriptionally activates ACAT2, stimulating cholesterol biosynthesis and promoting tumor cell proliferation while suppressing apoptosis.

We analyzed clinical data from The Cancer Genome Atlas (TCGA) melanoma cohort. ACAT2 expression was significantly elevated in melanoma tissues compared to normal skin samples (Fig. 6a; Supplementary Fig. S6a). Kaplan-Meier survival analysis revealed that high ACAT2 expression correlated with poor prognosis, indicating its value as a prognostic biomarker in melanoma (Fig. 6b). In clinical melanoma samples from the DFCI (Dana-Farber Cancer Institute) cancer database, ACAT2 expression exhibits a statistically significant, moderate positive correlation with SOX10 mRNA levels (Fig. 6c). Immunofluorescence staining of clinical samples confirmed that both SOX10 and ACAT2 proteins were highly expressed and co-localized in melanoma tissues compared to normal skin (Supplementary Fig. S6c).

When assessing ACAT2 expression across melanoma stages, we observed a pronounced increase in early-stage disease. Although ACAT2 levels remained elevated relative to normal tissue throughout progression, they did not significantly rise with advancing stage (Supplementary Fig. S6b). These findings suggest that the SOX10-ACAT2 axis contributes to melanoma pathogenesis, with ACAT2 potentially playing a more critical role in early tumor development.

In summary, we propose a model in which SOX10 recruits and together with TAF15 to transcriptionally activate ACAT2, enhancing *de novo* cholesterol synthesis and promoting melanoma proliferation. Unlike normal melanocytes, melanoma cells accumulate cholesterol, which suppresses apoptosis and stimulates proliferation. This SOX10-TAF15-ACAT2 regulatory axis thus represents a key mechanism driving early melanoma progression (Fig. 6d).

## 4. Discussion

Our study demonstrates that cholesterol accumulates in melanoma cells, and that activation of the cholesterol synthesis pathway inhibits apoptosis and promotes proliferation. SOX10—a transcription factor critical for melanocyte development and highly expressed in melanoma—positively regulates both cholesterol accumulation and *de novo* synthesis in melanoma cells. Specifically, SOX10 exerts a strong regulatory effect on ACAT2, a key enzyme in the cholesterol synthesis pathway. Mechanistically, SOX10 recruits TAF15 to form a transcriptional complex that enhances ACAT2 expression. We further showed that the SOX10-TAF15 axis modulates cholesterol levels in melanoma cells via ACAT2, thereby influencing apoptosis and proliferation. Using *in vivo* models, we confirmed that ACAT2, acting downstream of SOX10, regulates cholesterol content and tumor growth. Analysis of clinical melanoma samples revealed that high ACAT2 expression correlates with poor patient prognosis, and that SOX10 and ACAT2 levels are significantly associated. These findings collectively highlight the importance of the SOX10-TAF15-ACAT2-cholesterol axis in melanoma proliferation.

The role of cholesterol in cancer progression and the therapeutic potential of modulating its homeostasis remain controversial. While epidemiological studies have linked high circulating cholesterol levels to increased risks of prostate and colorectal cancers—and cholesterol-lowering interventions have shown preventive and therapeutic benefits (43, 44—other reports found no consistent association between serum cholesterol or statin use and cancer incidence. Overall, given the perplexing causal relationship between serum cholesterol and cancer progression, it is increasingly recognized that intracellular cholesterol levels in cancer cells, rather than dietary intake or serum cholesterol content, play a crucial role in tumor initiation and development 15. Elevated circulatory cholesterol enriches lipid rafts, altering downstream signaling pathways and promoting tumor growth 45. Cancer cells rely on heightened cholesterol metabolism to sustain rapid proliferation, membrane integrity, and signal transduction 46. Oncogenic processes often upregulate de novo cholesterol synthesis to meet these demands 47. From an immunometabolic perspective, inhibiting cholesterol synthesis or enhancing efflux can exert antitumor effects. For instance, ABCG1 deficiency in myeloid cells promotes M1 macrophage polarization, leading to direct tumor cell killing 48. SOAT1 knockdown in T cells reduces cholesteryl ester storage, enhances T-cell receptor clustering, and strengthens antitumor cytotoxicity against melanoma 10. Cholesterol depletion using methyl-β-cyclodextrin (MCD) sensitizes tumor cells to tamoxifen 49, while PTEN loss-driven cholesterol accumulation via AKT activation promotes prostate cancer growth—an effect reversible by cholesterol blockade 33. Similarly, upregulation of cholesterol synthesis has been shown to support proliferation and self-renewal in p53-mutant breast cancer models 50, 51. Collectively, these studies underscore that disrupting cholesterol homeostasis—whether through synthesis inhibition, transport blockade (e.g., via leelamine in melanoma 20, 52), or efflux promotion—can inhibit tumor progression. Our study further reveals that ACAT2, the catalytic enzyme responsible for the first committed step of cholesterol synthesis, acts as a key regulator of melanoma proliferation both in vitro and in vivo. Along with previously known cholesterol-metabolizing enzymes implicated in cancer, such as HMGCR, SOAT1, and ABCG1, ACAT2 represents a potential therapeutic target within the cholesterol synthesis pathway.

Research on transcription factors regulating cholesterol anabolism has predominantly focused on SREBP2, which directly controls multiple cholesterol metabolic enzymes 53, 54. Activation of the PI3K/AKT pathway elevates intracellular cholesterol by stimulating SREBP2, thereby promoting cholesterol synthesis, enhancing LDL receptor-mediated uptake, and suppressing ABCA1-mediated efflux. TP53 has also been reported to activate cholesterol synthesis 15. In this study, we identified SOX10 as a key transcription factor regulating ACAT2 expression, which in turn influences the cholesterol synthesis pathway. Although our analysis centered on ACAT2—the most markedly altered cholesterol anabolic enzyme following SOX10 modulation in melanoma cells—we also observed that SOX10 regulates other enzymes, including HMGCR, HMGCS1, IDI1, FDPS, SQLE, and SOAT1. There are still many contents that need to be further studied for the regulation of cholesterol metabolism and its related metabolic enzymes by SOX10. Notably, SOX10 is well established as a master regulator of neural crest development and melanoma differentiation 55, 56, with broad transcriptional influence extending beyond ACAT2. Thus, while our data support a direct SOX10-ACAT2-cholesterol axis, cholesterol alterations may also arise indirectly through SOX10-mediated changes in transcriptional programs or cellular states. For instance, SOX10 loss has been associated with invasive or extracellular matrix-enriched phenotypes 57, and promoter hypermethylation-induced silencing of SOX10 and other lineage genes can drive melanoma dedifferentiation 58. Such state transitions often involve remodeling of membrane lipids, membrane protein composition, and overall metabolic reprogramming, which may secondarily reshape cholesterol homeostasis. Thus, the observed increase in cholesterol synthesis may reflect both direct regulation via ACAT2 and secondary effects of SOX10-mediated differentiation control. Future studies integrating metabolic profiling with differentiation markers will be required to disentangle these contributions and to define the multifaceted role of SOX10 in coordinating cholesterol metabolism in melanoma.

While SOX10 acts as a classical transcription factor for the regulation of downstream genes, we found its binding to TAF15 and co-regulation of ACAT2 transcription, which has an important role in further expanding our inherent perception of SOX10. It is well known that TAF15 is a TATA box-binding protein that activates downstream gene expression by binding to the DNA region of gene promoter. SOX10 binding to TAF15 may illustrate that SOX10 also plays a role in the regulation of gene expression influenced by TAF15. Of course, these hypotheses are not yet supported by available research evidence, but it providesan interesting entry point for our further study. It should be noted, however, that while we demonstrate SOX10-TAF15 complex formation and its role in ACAT2 transactivation, our study does not establish whether TAF15 binds directly to the ACAT2 promoter or whether its binding sites overlap with those of SOX10. This represents a limitation of the current work and an important avenue for further investigation.

## Supplementary Material

Supplementary figures and tables.
