# Supplementary material for: The SOX10-ACAT2-Cholesterol Synthesis Axis Is Required for Melanoma Proliferation
Source: Int J Biol Sci. 2026 Jan 15;22(4):1717–32. doi: 10.7150/ijbs.114084 (PMC12905572; doi:10.7150/ijbs.114084)
Supplement: Supplementary file 1 — Supplementary figures and tables. [file ijbsv22p1717s1.pdf]

a

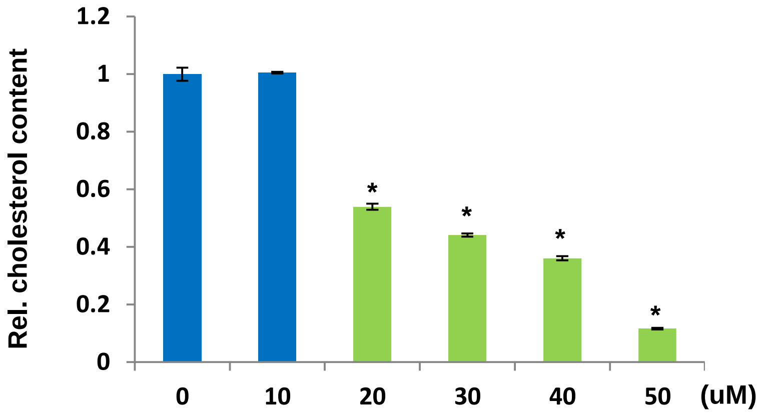

Supplementary Fig. S1 Avasimibe reduces total cholesterol levels in SK-MEL-28 cells.

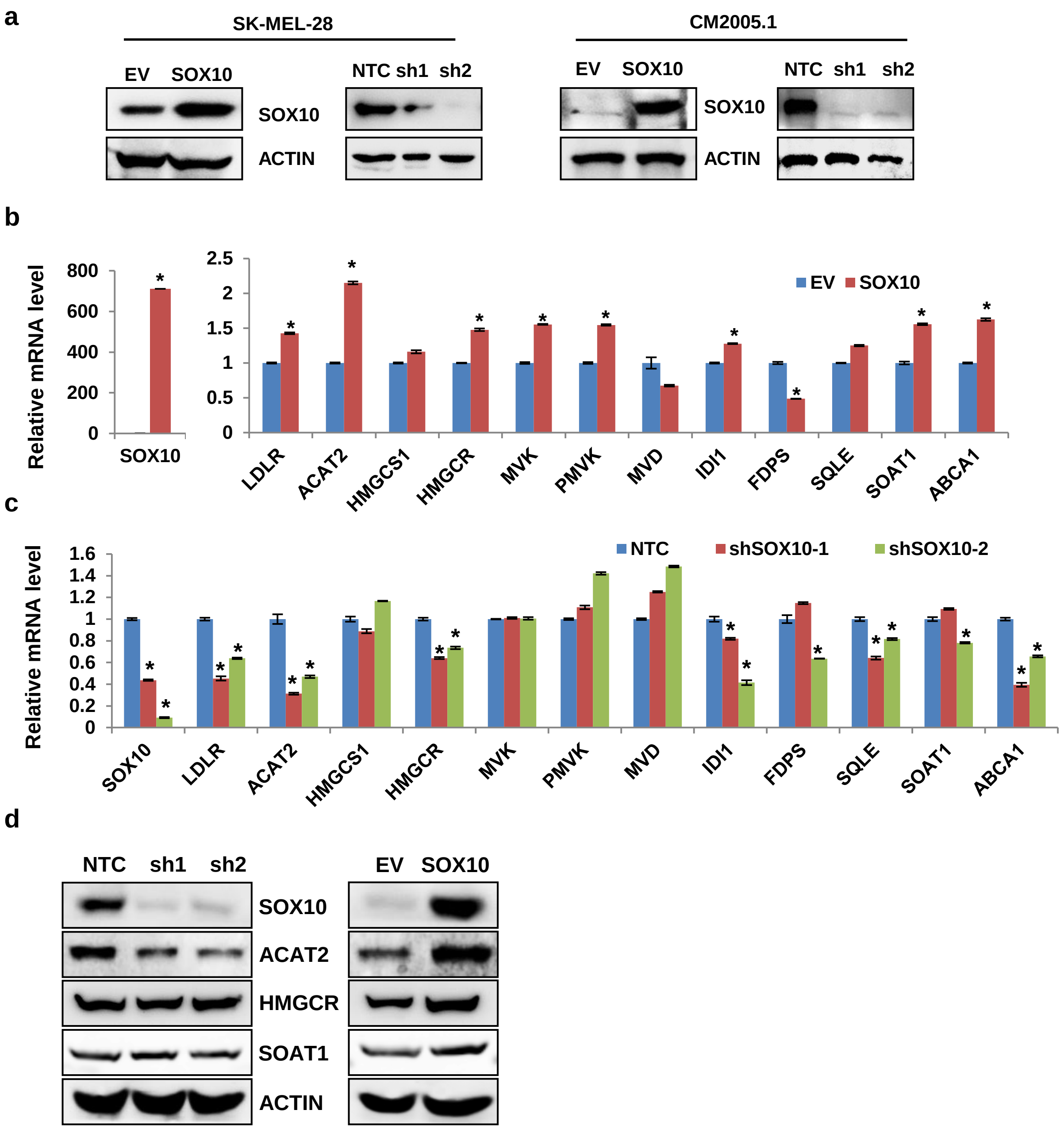

Supplementary Fig. S2 SOX10 upregulates ACAT2 expression.

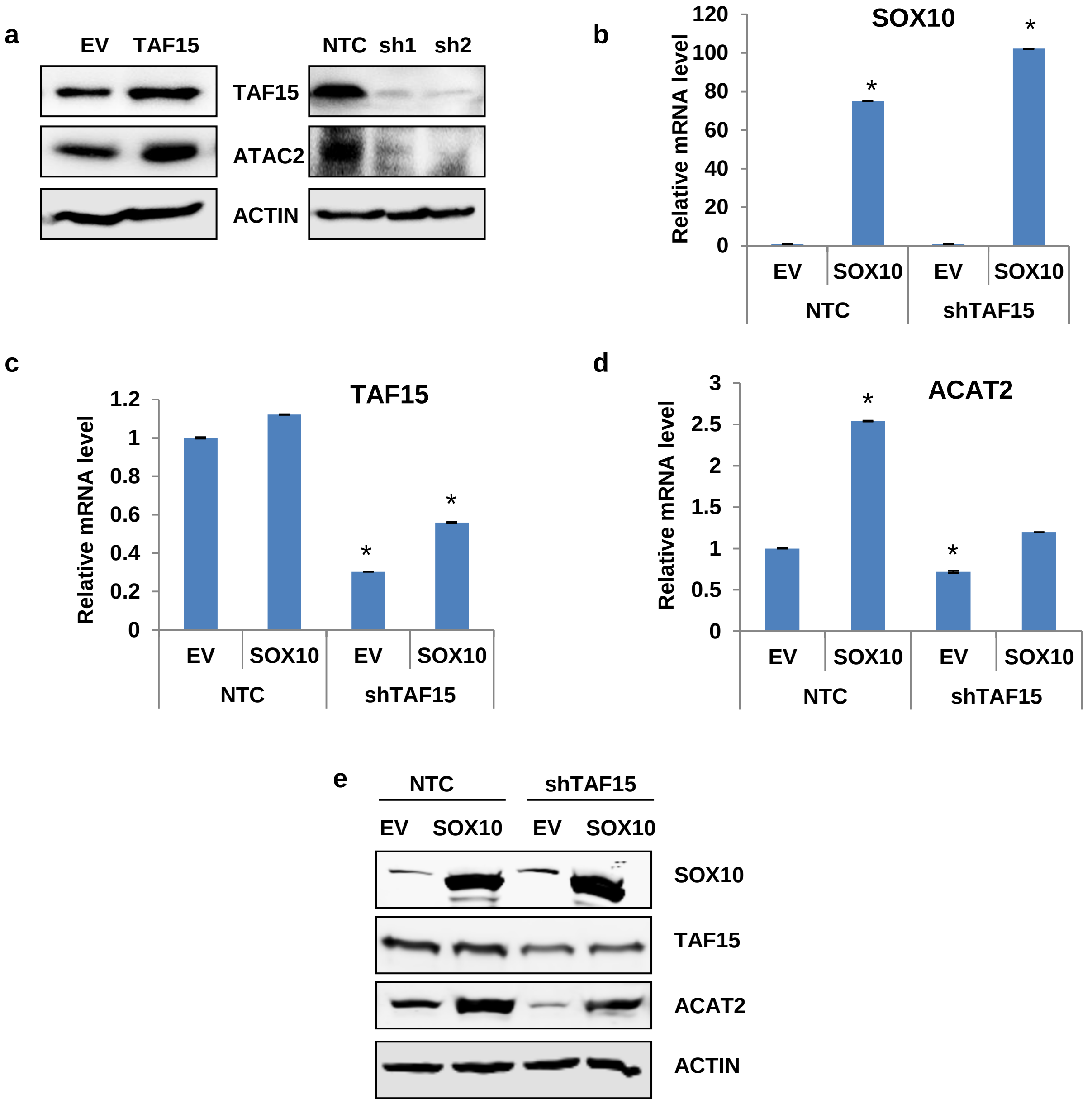

Supplymental Fig. S3 TAF15 positively regulates ACAT2 in melanoma cells.

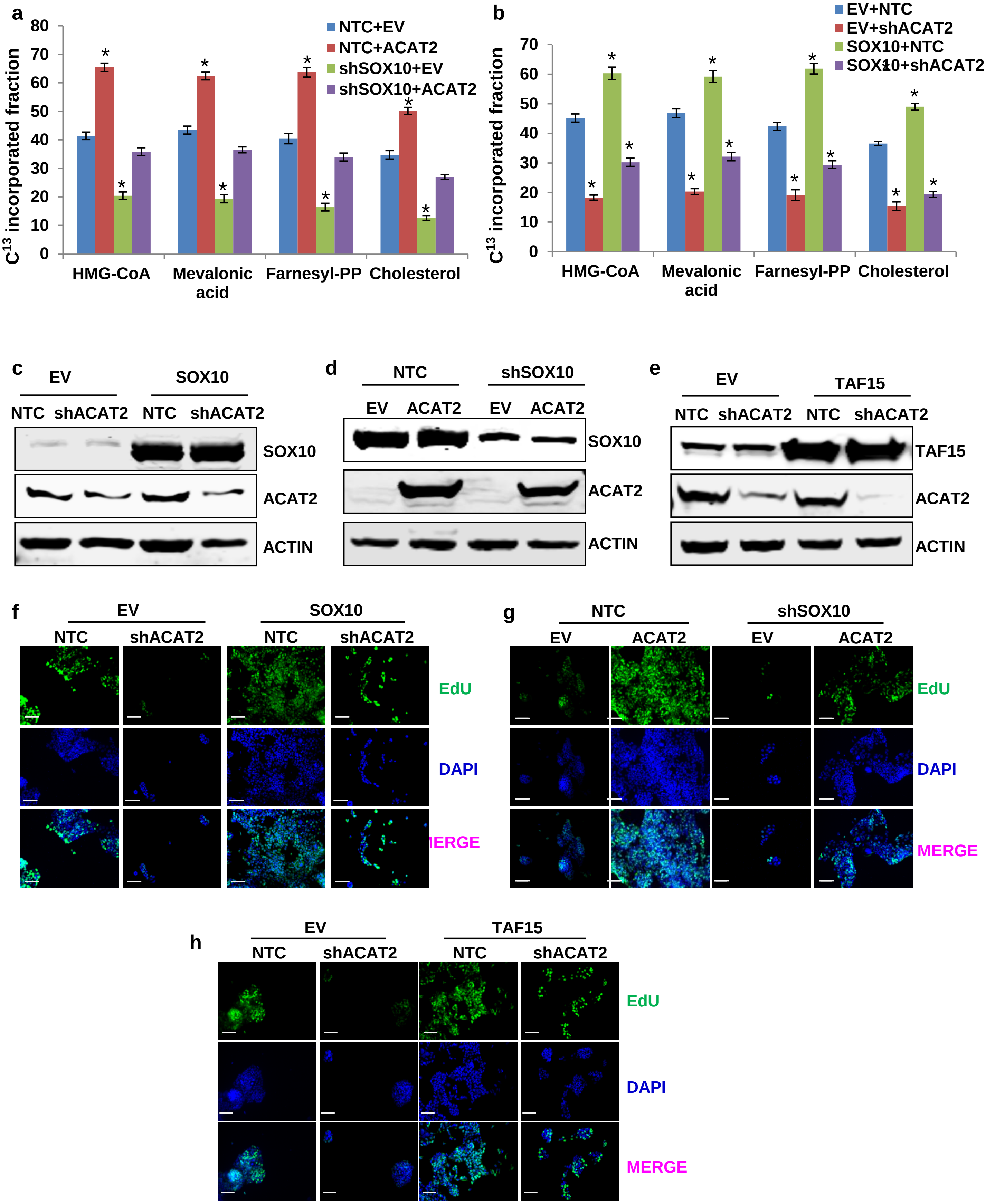

Supplymental Fig. S4 SOX10 promotes cholesterol synthesis via ACAT2 in melanoma cells.

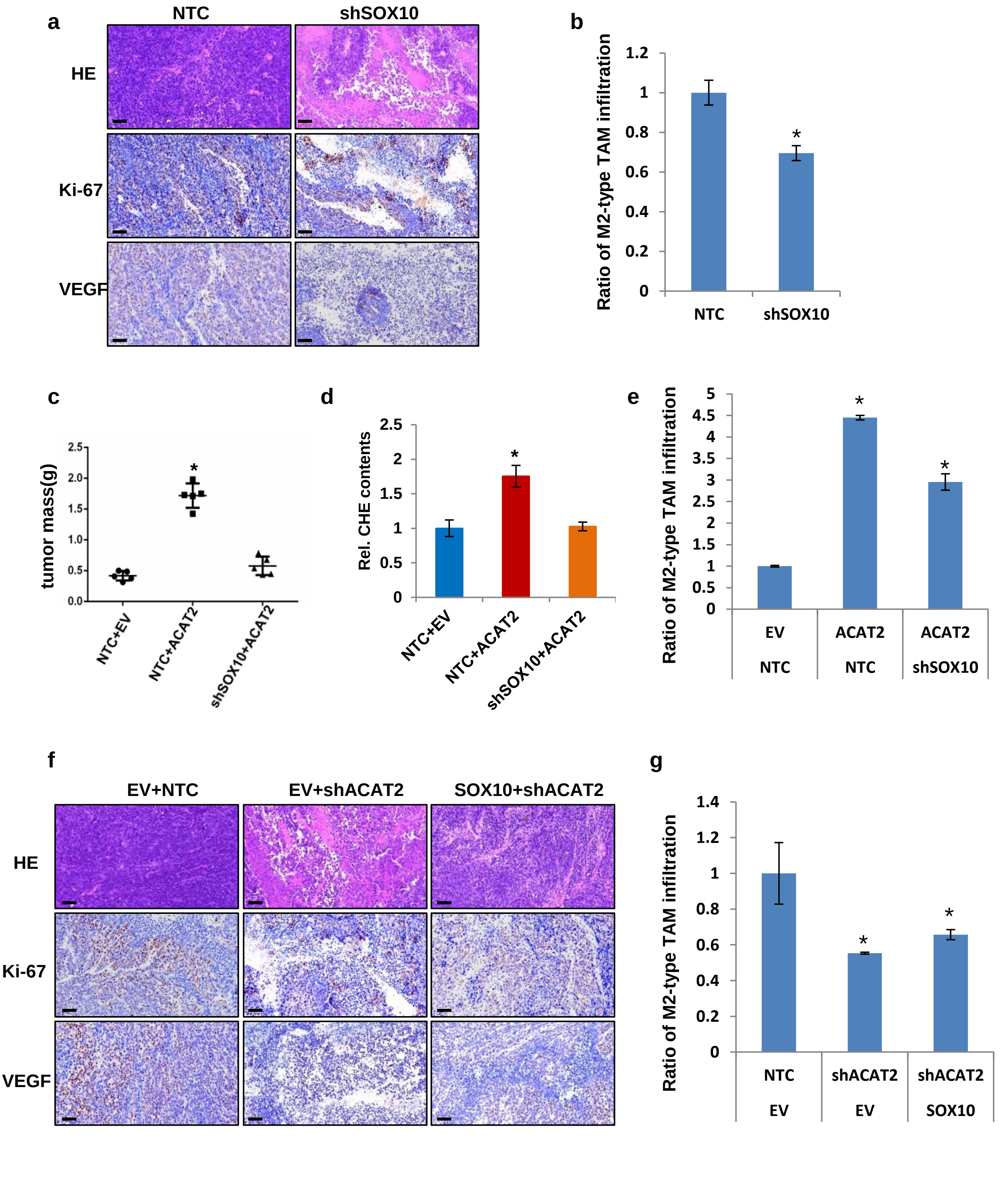

Supplymental Fig. S5 ACAT2 is essential for SOX10-driven melanoma progression *in vivo*.

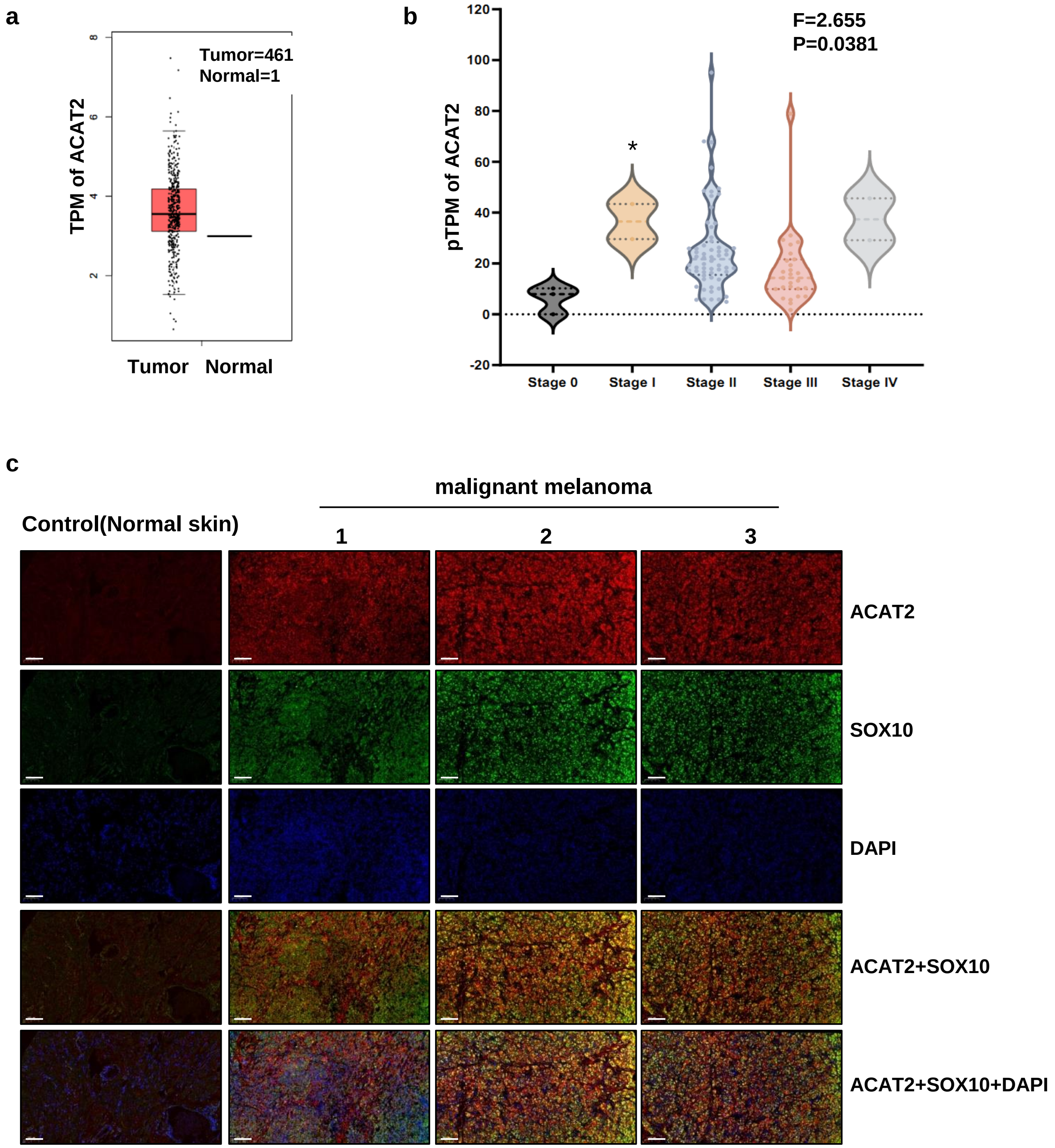

Supplymental Fig. S6 The SOX10-ACAT2 axis correlates with poor prognosis in human melanoma.

Supplementary information

The SOX10-ACAT2-Cholesterol Synthesis Axis Is Required for Melanoma Proliferation

Supplementary Fig. S1 (related to Fig. 1). Avasimibe reduces total cholesterol levels in SK-MEL-28 cells.

(a) Cellular cholesterol content was measured in SK-MEL-28 cells treated with 0–50 μM avasimibe and normalized to total protein. Data represent mean ± SD of three independent experiments. *P < 0.05 versus the 0 μM group by one-way ANOVA with Bonferroni post-hoc test.

Supplementary Fig. S2 (related to Fig. 2). SOX10 upregulates ACAT2 expression.

(a) Western blot analysis of SOX10 expression in SK-MEL-28 and CM2005.1 cells with SOX10 overexpression or knockdown.

(b, c) qRT-PCR analysis of cholesterol metabolism-related genes in CM2005.1 cells with SOX10 overexpression **(b)** or knockdown **(c).**

(d) Western blot analysis of SOX10 expression in CM2005.1 cells with SOX10 overexpression or knockdown.

Data are presented as mean ± SD from three independent experiments. *P < 0.05 compared to the EV group in (b) or the NTC group in (c) by one-way ANOVA with Bonferroni post-hoc test. Actin served as a loading control in (a, d).

Supplementary Fig. S3 (related to Fig. 3). TAF15 positively regulates ACAT2 in melanoma cells.

(a) Western blot analysis of ACAT2 and TAF15 expression in CM2005.1 cells with TAF15 overexpression or knockdown.

(b–d) qRT-PCR analysis of SOX10 **(b)**, TAF15 **(c)**, and ACAT2 **(d)** expression in SK-MEL-28 cells with SOX10 overexpression, with or without TAF15 knockdown.

(e) Western blot analysis of SOX10, TAF15, and ACAT2 protein levels under the same conditions as in (b–d).

Data represent mean ± SD; n = 3 independent experiments. *P < 0.05 versus the EV or NTC group in (b–d) by one-way ANOVA with Bonferroni post-hoc test. Actin served as a loading control in (a, e).

Supplementary Fig. S4 (related to Fig. 4). SOX10 promotes cholesterol synthesis via ACAT2 in melanoma cells.

(a) LC-MS analysis of ^{13}C-labeled HMG-CoA, mevalonic acid, farnesyl-PP, and cholesterol in SK-MEL-28 cells expressing NTC or shSOX10, with or without ACAT2 overexpression, after 48 h incubation with 10 mM ^{13}C-acetate.

(b) LC-MS analysis of ^{13}C-labeled metabolites (as in a) in SK-MEL-28 cells expressing EV or SOX10, with or without ACAT2 knockdown.

(c-e) Western blot analysis of ACAT2 and SOX10 in SK-MEL-28 cells with SOX10 overexpression plus ACAT2 knockdown **(c)**, SOX10 knockdown plus ACAT2 overexpression **(d)**, or TAF15 overexpression plus ACAT2 knockdown **(e)**.

(f-h) EdU staining of SK-MEL-28 cells with SOX10 overexpression ± ACAT2 knockdown **(f)**, SOX10 knockdown ± ACAT2 overexpression (g),

or TAF15 overexpression ± ACAT2 knockout **(h).**

Data represent mean ± SD; n = 3. *P < 0.05 versus the respective EV or NTC group in (a, b) by one-way ANOVA with Bonferroni post-hoc test. Actin served as a loading control in (c-e). Scale bars: 100 μm (f-h).

Supplementary Fig. S5 (related to Fig. 5). ACAT2 is essential for SOX10-driven melanoma progression *in vivo*.

(a, b) SK-MEL-28 cells stably expressing NTC or shSOX10 were subcutaneously injected into nude mice. Frozen sections of tumor xenografts were subjected to hematoxylin and eosin (H&E) staining, Ki-67 staining for proliferation assessment, and VEGF staining for angiogenesis evaluation **(a)**. Flow cytometry analysis of the proportion of F4/80$^+$CD11b$^+$ tumor-associated macrophages (TAMs) in the collected xenografts **(b)**.

(c, d) SK-MEL-28 cells stably expressing NTC or shSOX10, with or without ACAT2 overexpression, were subcutaneously injected into nude mice (n = 5 per group). Tumor weights at the endpoint **(c)**. Cellular cholesterol content measured in five independent tumors per group and normalized to total protein **(d)**.

(e) Flow cytometry analysis of F4/80$^+$CD11b$^+$ TAMs in xenografts from mice injected with SK-MEL-28 cells expressing NTC or shSOX10 plus

ACAT2 overexpression.

(f, g) SK-MEL-28 cells with ACAT2 knockdown, with or without SOX10 overexpression, were subcutaneously injected into nude mice. H&E, Ki-67, and VEGF staining of tumor sections **(f)**. Flow cytometry analysis of F4/80^{+}CD11b^{+} TAMs **(g)**.

Data are presented as mean ± SD. *P < 0.05 versus the NTC group in (b) or the EV/NTC groups in (c–e, g) by one-way ANOVA with Bonferroni post-hoc test. n = 3 (b, e, g); n = 5 (c, d). Scale bars: 50 μm (a, f).

Supplementary Fig. S6 (related to Fig. 6). The SOX10-ACAT2 axis correlates with poor prognosis in human melanoma.

(a) ACAT2 expression (TPM) in melanoma and normal tissues from TCGA.

(b) Association between ACAT2 expression and melanoma pathological stage (TCGA).

(c) Immunofluorescence of ACAT2 (red) and SOX10 (green) in normal skin and melanoma tissues from toe (1), orbit (2), and sole (3). Nuclei were stained with DAPI (blue). Scale bars: 100 μm.

Supplementary Table 1 (related to Fig. 1). Catalytic enzymes involved in cholesterol metabolism.

**Abbreviations**	**Full name of the catalytic enzyme**	**briefly describing of catalytic functions**
**LDLR**	low-density lipoprotein receptor	Mediates receptor-mediated endocytosis of cholesterol-rich LDL particles.
**ACAT1**	acetyl-coA acetyltransferase 1	Tetrameric enzyme in the ketogenesis pathway; condenses two acetyl-CoA molecules to acetoacetyl-CoA.
**ACAT2**	acetyl-coA acetyltransferase 2	Catalyzes the first committed step of cholesterol synthesis: condensation of two acetyl-CoA to acetoacetyl-CoA.
**HMGCS1**	3-hydroxy-3-methylglutaryl-CoA synthase 1	Condenses acetoacetyl-CoA with acetyl-CoA to form HMG-CoA in the mevalonate pathway.
**HMGCR**	3-hydroxy-3-methylglutaryl-CoA reductase	Rate-limiting enzyme of cholesterol synthesis; reduces HMG-CoA to mevalonate.
**MVK**	mevalonate kinase	Phosphorylates mevalonate to mevalonate-5-phosphate in the mevalonate pathway.
**PMVK**	phosphomevalonate kinase	Phosphorylates mevalonate-5-phosphate to mevalonate-5-diphosphate.
**MVD**	mevalonate diphosphate decarboxylase	Decarboxylates mevalonate-5- diphosphate to isopentenyl diphosphate (IPP).
**IDI1**	isopentenyl-diphosphate delta-isomerase	Isomerizes IPP to dimethylallyl diphosphate (DMAPP).
**FDPS**	farnesyl diphosphate synthase	Condenses IPP with DMAPP to form geranyl diphosphate, then farnesyl diphosphate (FPP).
**SQLE**	Squalene epoxidase	Catalyzes the first oxygenation step in cholesterol synthesis: conversion of squalene to 2,3-oxidosqualene.
**SOAT1**	sterol-o-acyltransferase 1 (also named acyl-coA cholesterol acyltransferase 1)	Integral membrane protein of the rough ER; esterifies cholesterol with long-chain fatty acids to form cholesteryl esters.
**ABCA1**	ATP-binding cassette transporter A1	Mediates cholesterol and phospholipid efflux to apolipoprotein A-I for HDL assembly; regulates cellular cholesterol homeostasis.

Supplementary Table 2 (related to Fig. 3). List of SOX10-interacting candidate proteins identified by mass spectrometry.

Accession	Description	Score	Coverage	# Proteins	# Unique Peptides	# Peptides	# PSMs	Area	# AAs	MW [kDa]	calc. pI
K7EP68	TPM4	15.36	35.51	43	4	4	6	4.095E7	138	15.8	5.00
	A2	Sequence	# PSMs	# Proteins	# Protein Groups	Protein Group Accessions	Modifications	ΔCn	Area	XCorr	Charge
	High	LEEAEKAADESER	2	39	1	K7EP68		0.0000	2.225E7	2.86	2
	High	HIAEEADRKYEEVAR	1	17	1	K7EP68		0.0000	1.574E7	2.83	4
	High	IQLVEEELDRAQER	1	39	1	K7EP68		0.0000	8.486E7	2.63	3
	High	LATALQK	2	40	1	K7EP68		0.0000	8.558E6	2.24	2
P35579	MYH9	14.93	3.83	19	6	6	6	3.489E7	1960	226.4	5.60
	A2	Sequence	# PSMs	# Prote	# Protei	Protein Group Accessions	Modifications	ΔCn	Area	XCorr	Charge

				ins	n Groups						
	High	KQELEEIcHDLEAR	1	2	1	P35579	C8(Carbamidomethyl)	0.0000	2.710E7	3.07	3
	High	KFDQLLAEEK	1	17	1	P35579		0.0000	4.392E7	2.49	2
	High	QAQQERDELADEIANSSGK	1	2	1	P35579		0.0000	3.086E7	2.44	3
	High	KEEELQAALAR	1	12	1	P35579		0.0000	2.892E7	2.40	2
	High	QLEEAEEEAQR	1	2	1	P35579		0.0000	1.467E7	2.34	2
	High	TDLLLEPYNK	1	2	1	P35579		0.0000	2.988E7	2.19	2
P60709	Actin	9.76	12.00	30	3	3	3	1.525E8	375	41.7	5.48
	A2	Sequence	# PSMs	# Proteins	# Protein Groups	Protein Group Accessions	Modifications	ΔCn	Area	XCorr	Charge
	High	VAPEEHPVLLTEAPLNPK	1	9	1	P60709		0.0000	2.861E8	3.75	3
	High	SYELPDGQVITIGNER	1	14	1	P60709		0.0000	1.574E8	3.12	2

	High	AVFPSIVGRPR	1	28	1	P60709		0.0000	1.395E7	2.89	3
P56693-2	SOX-10	6.91	37.76	1	9	9	9	1.010E8	286	31.1	7.68
	A2	Sequence	# PSMs	# Proteins	# Protein Groups	Protein Group Accessions	Modifications	ΔCn	Area	XCorr	Charge
	High	EQQDGEADDDKFPVcIR	1	1	1	P56693-2	C15(Carbamidomethyl)	0.0000	8.797E7	3.32	3
	High	DHPDYK	1	1	1	P56693-2		0.0000	8.725E6	1.88	2
	High	TELQSGK	1	1	1	P56693-2		0.0000	0.000E0	1.70	2
	High	LLNESDKRPFIEEAER	1	1	1	P56693-2		0.0000	4.424E7	1.39	4
	High	ASPGPGELGK	1	1	1	P56693-2		0.0000	7.321E6	1.30	2
	High	LADQYPHLHNAELSK	1	1	1	P56693-2		0.0000	3.219E7	1.19	4
	High	SAHLDHR	1	1	1	P56693-2		0.0000	1.780E7	1.14	2
	High	AAQGEAEcPGGEAEQGGTAAIQAHYK	1	1	1	P56693-2	C8(Carbamidomethyl)	0.0000	1.709E8	1.12	3
	High	TELQSGKADPK	1	1	1	P56693-2		0.000	4.743	1.07	2

								0	E6		
Q92804-2	TAF15	5.45	8.49	5	1	2	2	6.511E7	589	61.5	8.02
	A2	Sequence	# PSMs	# Proteins	# Protein Groups	Protein Group Accessions	Modifications	ΔCn	Area	XCorr	Charge
	High	SGGGYGGDR	1	4	1	Q92804-2		0.0000	1.258E7	2.94	2
	High	GEATVSFDDPPSAK	1	6	2	P35637-2;Q92804-2		0.0000	1.177E8	2.51	2
P35637-2	FUS	5.12	3.05	4	1	2	2	1.546E8	525	53.3	9.36
	A2	Sequence	# PSMs	# Proteins	# Protein Groups	Protein Group Accessions	Modifications	ΔCn	Area	XCorr	Charge
	High	LKGEATVSFDDPPSAK	1	3	1	P35637-2		0.0000	1.915E8	2.62	3
	High	GEATVSFDDPPSAK	1	6	2	P35637-2;Q92804-2		0.0000	1.177E8	2.51	2
F8VPF3	MYL6	4.81	21.54	13	2	2	2	5.243E8	130	14.4	4.51
	A2	Sequence	# PSMs	#	#	Protein Group	Modifications	ΔCn	Area	XC	Char

				Proteins	Protein Groups	Accessions				orr	ge
	High	NKDQGTYEDYVEGLR	1	11	1	F8VPF3		0.0000	3.354E8	2.47	3
	High	ALGQNPTNAEVLK	1	10	1	F8VPF3		0.0000	7.132E8	2.34	2
C9JKR2	Albumin	4.11	3.60	6	1	1	1	3.530E8	417	47.3	6.35
	A2	Sequence	# PSMs	# Proteins	# Protein Groups	Protein Group Accessions	Modifications	ΔCn	Area	XCorr	Charge
	High	KVPQVSTPTLVEVSR	1	6	1	C9JKR2		0.0000	3.530E8	4.11	3
A6PVD3	SOX10	3.32	7.98	3	1	1	1	8.797E7	213	23.3	7.53
	A2	Sequence	# PSMs	# Proteins	# Protein Groups	Protein Group Accessions	Modifications	ΔCn	Area	XCorr	Charge
	High	EQQDGEADDDKFPVcIR	1	3	1	A6PVD3	C15(Carbamidomethyl)	0.0000	8.797E7	3.32	3

Q86V81	ALYREF	2.91	4.28	2	1	1	1	5.672E6	257	26.9	11.15
	A2	Sequence	# PSMs	# Proteins	# Protein Groups	Protein Group Accessions	Modifications	ΔCn	Area	XCorr	Charge
	High	SLGTADVHFER	1	2	1	Q86V81		0.0000	5.672E6	2.91	3
P81605	Dermcidin	2.55	10.00	2	1	1	1	4.617E7	110	11.3	6.54
	A2	Sequence	# PSMs	# Proteins	# Protein Groups	Protein Group Accessions	Modifications	ΔCn	Area	XCorr	Charge
	High	ENAGEDPGLAR	1	2	1	P81605		0.0000	4.617E7	2.55	2
J3KTJ1	MYL12A	2.54	9.65	5	1	1	1	7.709E7	114	13.0	4.88
	A2	Sequence	# PSMs	# Proteins	# Protein Groups	Protein Group Accessions	Modifications	ΔCn	Area	XCorr	Charge
	High	LNGTDPEDVIR	1	5	1	J3KTJ1		0.000	7.709	2.54	2

								0	E7		
Q5JVS8	Vimentin	2.52	5.78	4	1	1	1	1.268 E7	173	20.0	4.89
	A2	Sequence	# PSMs	# Proteins	# Protein Groups	Protein Group Accessions	Modifications	ΔCn	Area	XCorr	Charge
	High	QDVDNASLAR	1	4	1	Q5JVS8		0.0000	1.268 E7	2.52	2
Q86YZ3	Hornerin	2.52	1.68	1	1	1	1	7.310 E6	2850	282.2	10.04
	A2	Sequence	# PSMs	# Proteins	# Protein Groups	Protein Group Accessions	Modifications	ΔCn	Area	XCorr	Charge
	High	GPYESGSGHSSGLGHR	1	1	1	Q86YZ3		0.0000	7.310 E6	2.52	3
F8WBR5	Calmodulin	2.44	26.15	7	1	1	1	2.855 E8	65	7.4	4.01
	A2	Sequence	# PSMs	# Proteins	# Protein Groups	Protein Group Accessions	Modifications	ΔCn	Area	XCorr	Charge

	High	EAFSLFDKDGDGTITTK	1	7	1	F8WBR5		0.0000	2.855E8	2.44	3
F6UXX1	SYNCRIP	2.32	5.41	5	1	1	1	1.512E7	185	20.2	4.93
	A2	Sequence	# PSMs	# Proteins	# Protein Groups	Protein Group Accessions	Modifications	ΔCn	Area	XCorr	Charge
	High	DSDLSHVQNK	1	5	1	F6UXX1		0.0000	1.512E7	2.32	2
P01617	Ig kappa chain V-II region	2.30	6.19	3	1	1	1	2.447E7	113	12.3	6.00
	A2	Sequence	# PSMs	# Proteins	# Protein Groups	Protein Group Accessions	Modifications	ΔCn	Area	XCorr	Charge
	High	ASGVPDR	1	3	1	P01617		0.0000	2.447E7	2.30	2
P62805	Histone H4	2.25	11.65	1	1	1	1	7.222E6	103	11.4	11.36
	A2	Sequence	# PSMs	#	#	Protein Group	Modifications	ΔCn	Area	XC	Char

				Proteins	Protein Groups	Accessions				orr	ge
	High	DNIQGITKPAIR	1	1	1	P62805		0.0000	7.222E6	2.25	3
Q01105-3	SET	2.19	4.89	5	1	1	1	3.200E7	266	31.1	4.23

Supplementary Table 3. Nucleotide Sequences of Primers Used for qRT-PCR.

Gene	species	sequence of forward primer	sequence of reverse primer
LDLR	human	ACGGCGTCTCTTCCTATGACA	CCCTTGGTATCCGCAACAGA
ACAT2	human	CTTTAGCACGGATAGTTTCCTGG	GCTGCAAAGGCTTCATTGATTTC
HMGCS1	human	CATTAGACCGCTGCTATTCTGTC	TTCAGCAACATCCGAGCTAGA
HMGCR	human	TGATTGACCTTTCCAGAGCAAG	CTAAAATTGCCATTCCACGAGC
MVK	human	GGAGCAAGGTGATGTCACAAC	CGGCAGATGGACAGGTATAAGT
PMVK	human	CCTTTCGGAAGGACATGATCC	TCTCCGTGTGTCACTCACCA
MVD	human	GGACCGGATTTGGCTGAATG	CCCATCCCGTGAGTTCCTC
IDI1	human	TCCATTAAGCAATCCAGCCGA	CCCAGATACCATCAGACTGAGC
FDPS	human	TGTGACCGGCAAAATTGGC	GCCCGTTGCAGACACTGAA
SQLE	human	TGACAATTCTCATCTGAGGTCCA	CAGGGATACCCTTTAGCAGTTTT
Soat1	human	CAAGGCGCTCTCTCTTAGATG	GGTCCAAACAACGGTAGGAAA
ABCA1	human	TTCCCGCATTATCTGGAAAGC	CAAGGTCCATTTCTTGGCTGT
ACAT1	human	AGGCTGGTGCAGGAAATAAGATATG	ATAGGTAAGCCTGCACCCAA
TAF15	human	GATTCTGGAAGTTACGGTCAGTC	AGCTTTGTGATGCTTGTCCATAG

SOX10	human	CCTCACAGATCGCCTACACC	CATATAGGAGAAGGCCGAGTAGA

Supplementary Table 4. Sequences of shRNAs used in this study

target gene name	accession number	sequence of forward oligo	sequence of reverse oligo
shACAT2-1	NM_005891.2	CCGGTCAGAGAGAATGAATTGCTTACTCGAGTAAGCAATTCATTCTCTCTGATTTTTG	AATTCAAAAATCAGAGAGAATGAATTGCTTACTCGAGTAAGCAATTCATTCTCTCTGA
shACAT2-2	NM_005891.3	CCGGCCAGCCATAAAGCAAGCTGTTCTCGAGAACAGCTTGCTTTATGGCTGGTTTTTG	AATTCAAAAACCAGCCATAAAGCAAGCTGTTCTCGAGAACAGCTTGCTTTATGGCTGG
shTAF15-1	NM_003487.2	CCGGCCTATCATTCACAAAGGGAAACTCGAGTTTCCCTTTGTGAATGATAGGTTTTT	AATTCAAAAACCTATCATTCACAAAGGGAAACTCGAGTTTCCCTTTGTGAATGATAGG
shTAF15-2	NM_003487.2	CCGGGCAGCAAAGTTATTCTACCTACTCGAGTAGGTAGAATAACTTTGCTGCTTTTT	AATTCAAAAAGCAGCAAAGTTATTCTACCTACTCGAGTAGGTAGAATAACTTTGCTGC
shSOX10-1	NM_006941.3	CCGGCCTCATTCTTTGTCTGAGAAACTCGAGTTTCTCAGACAAAGAATGAGGTTTTTG	AATTCAAAAACCTCATTCTTTGTCTGAGAAACTCGAGTTTCTCAGACAAAGAATGAGG
shSOX10-2	NM_006941.3	CCGGGCAGCCAGTATATACGACACTCTCGAGAGTGTCGTATATACTGGCTGCTTTTTG	AATTCAAAAAGCAGCCAGTATATACGACACTCTCGAGAGTGTCGTATATACTGGCTGC

Supplementary Table 5 (related to Fig 3). Nucleotide Sequences of Primers Used for ChIP assay

target gene name	sequence of forward oligo	sequence of reverse oligo
ACAT2-primer1	AGAATCGCTTGAACCCGGGAG	TTTGAGACGGAGCTCGCTCTG
ACAT2-primer2	CATCGTGGCTCACTGCAACCT	AAATAAGCCGGGCATGGTGG
